# Deciphering lung adenocarcinoma heterogeneity: a multi-omics approach reveals nuclear division fibroblasts as prognosticators and therapeutic targets

**DOI:** 10.1186/s12967-026-08022-3

**Published:** 2026-03-20

**Authors:** Peng Cao, Yongxia Qin, Dingtao Hu, Tengfei Ge, Peng Zhang, Chao Cheng, Shun Wang, Shuhui Hu, Zhen Zhang, Ling Xu

**Affiliations:** 1 Department of Interventional Pulmonary Diseases, Anhui Chest Hospital, 397 Jixi Road, Hefei, 230022 China; 2 Blood Purification Center, The First Affiliated Hospital of Anhui Medical University, Hefei, 230022 China; 3 Department of Oncology, The First Affiliated Hospital of Anhui Medical University, Hefei, 230022 China; 4 Department of Thoracic Surgery, Anhui Chest Hospital, Hefei, 230022 China; 5 Department of Respiratory Medicine, Shanghai Xuhui Central Hospital, Zhongshan-Xuhui Hospital, Fudan University, Shanghai, 200031 China; 6 Huilong Town Health Center in Funan County, Fuyang, 236316 China

**Keywords:** Lung associated fibroblast, Lung adenocarcinoma, Single-cell RNA sequencing, Machine learning, Personalized therapy

## Abstract

**Background:**

Lung adenocarcinoma (LUAD) is a predominant contributor to cancer‑related mortality globally. Lung‑associated fibroblasts (LAFs) are intricately linked to tumorigenesis and the tumor microenvironment (TME), but their heterogeneity and prognostic relevance in LUAD remain incompletely understood. This study aimed to systematically characterize LAF subsets across the spectrum of pulmonary disease, identify LAF subpopulations associated with LUAD prognosis, and construct a robust LAF‑based prognostic signature.

**Methods:**

We employed a multi-omics approach, leveraging bulk RNA data of 2719 patients from 19 LUAD cohorts, single-cell RNA (scRNA) sequencing data of 368,904 cells from 93 samples, and spatial transcriptomics data of 15,673 spots from 6 samples to characterize the landscape of LAFs across various stages of pulmonary disease. We employed multiple advanced machine learning algorithms to construct and validate a robust nuclear division LAFs (nLAFs) risk score (nLRS) prediction model.

**Results:**

We observed a dynamic and gradual increase in the proportion of LAFs during the progression of LUAD. Throughout this process, we identified nine LAFs subtypes and found nLAFs are significantly associated with the prognosis of LUAD. Utilizing 100 machine learning algorithm combinations and integrating nLAFs marker genes, we developed a five gene based nLRS model, which demonstrated superior performance than other 49 published models in predicting clinical outcomes for LUAD. Additionally, we observed distinct biological functions and immune cell infiltration in the TME between high and low nLRS groups. Exploratory analysis of pan-cancer immunotherapy cohorts suggested that patients with high nLRS scores may exhibit resistance to immunotherapy in some cancer types, but prospective validation in LUAD-specific cohorts is required. Conversely, high nLRS patients displayed increased sensitivity to chemotherapeutic and targeted therapies in preclinical models.

**Conclusion:**

Our study introduces a candidate five-gene signature derived from nLAFs that may serve as a robust prognostic biomarker pending prospective validation, offering insights into personalized therapeutic strategies for LUAD patients.

**Supplementary Information:**

The online version contains supplementary material available at 10.1186/s12967-026-08022-3.

## Introduction

Lung adenocarcinoma (LUAD) remains the leading cause of cancer-related mortality worldwide [1]. Despite significant advancements in diagnostic techniques and therapeutic strategies, patient prognosis remains poor [2]. Although the promise shown by targeted and immunotherapies, the inherent heterogeneity of LUAD, along with challenges such as resistance and immune evasion, results in only a subset of patients benefiting from these interventions [3, 4]. Consequently, there is an urgent need for reliable biomarkers that can predict the prognosis, guiding personalized therapy strategies in LUAD, thereby enhancing patient outcomes.

Numerous studies have identified several molecular markers associated with LUAD prognosis [5–7]. While these markers have been extensively researched and can serve as prognostic indicators in certain contexts, their inconsistent performance across diverse patient populations restricts their widespread applicability [4, 7, 8]. Additionally, immune cell infiltration and stromal components within the tumor microenvironment (TME) are believed to be closely associated with prognosis [9, 10], the precise mechanisms underlying these relationships are not yet fully understood, resulting in only a fraction of patients benefiting from these insights [3, 5, 11]. Recent research has increasingly focused on therapies targeting TME components, such as tumor-associated macrophages (TAMs), dendritic cells (DCs), and cancer-associated fibroblasts (CAFs) [12–15]. Cancer-associated fibroblasts (CAFs), which are integral to solid tumors, have been linked to poor prognosis across various cancer types. Recently, single-cell RNA sequencing (scRNA-seq) and spatial transcriptomics RNA (st-RNA) sequencing have emerged as a powerful tool for investigating the heterogeneity of CAFs, revealing different cellular states, marker genes, and associated functions, thereby paving the way for personalized medicine [12, 15–18].

Recent pan-cancer studies have identified a proliferative CAF (pCAF) subtype characterized by cell cycle-related gene expression (e.g., STMN1, TOP2A, MKI67) across multiple solid tumors including pancreatic, ovarian, and lung cancers [15, 17, 19]. These pCAFs are thought to represent an activated, proliferating fibroblast state distinct from matrix-producing myofibroblastic CAFs (myCAFs) and inflammatory CAFs (iCAFs) [20]. However, whether pCAFs in lung adenocarcinoma possess distinct functional properties and clinical relevance compared to other cancer types remains incompletely understood.”

In this study, we analyzed scRNA-seq data from multiple samples spanning the full spectrum of pulmonary conditions, ranging from normal lung to LUAD, to elucidate the roles and functions of lung‑associated fibroblasts (LAFs) in lung disease development and progression. Subsequently, utilizing the Scissor algorithm, we noticed the nuclear division LAFs (nLAFs) is the most prognosis relevant. Based on the significant marker genes of nLAFs, we developed a nLAFs risk score (nLRS) model using the a comprehensive machine learning framework. Furthermore, we investigated the complex interplay between model genes, nLRS levels, and immune characteristics within LUAD. Additionally, we examined and validated the response of model genes and nLRS to immunotherapy and chemotherapy, aiming to provide further insights into personalized medicine for LUAD.

## Materials and methods

### Acquisition and preprocessing of the multi-omics data

This study utilizes scRNA-seq data from a curated set of 93 human lung samples to explore cellular diversity and temporal changes within the pulmonary microenvironment across various pathological stages, from normal lung tissue to LUAD [21, 22]. Additionally, RNA-sequencing (RNA-seq) expression profiles and clinical data were obtained from The Cancer Genome Atlas (TCGA) dataset (https://www.cancer.gov/ccg/research/genome-sequencing/tcga). We further enhanced our analysis with gene expression data and clinical details from three additional LUAD cohorts (GSE72094, GSE50081, and GSE31210) [23–25], as well as four independent immunotherapy cohorts, including IMvigor210, GSE135222, GSE78220 and GSE91061 [26–29]. st-RNA data from nine samples, including one normal (#HD), two normal tissues adjacent to tumor (#P10_B, #P16_B) and six LUAD tissues (#P10_T, #P11_T, #P16_T, #P17_T, #P24_T, and # P25_T), were retrieved from the E-MTAB-13530 cohort [30]. Drug sensitivity data were sourced from the Genomics of Drug Sensitivity in Cancer (GDSC) database [31]. The characteristics of the multi-omic data used in the current study can be found in Table S1. Additionally, to further elucidate the characteristics and heterogeneity of CAFs across different cancer types and to validate our initial single-cell atlas, we conducted a pan-cancer analysis. This analysis encompassed 226 samples from 10 solid tumor types, sourced from the GSE210347 dataset [32]. Furthermore, we included an additional 42 samples from an independent LUAD cohort (GSE148071) [33] to specifically validate our findings in lung adenocarcinoma. This comprehensive approach allowed us to compare CAFs across a broad spectrum of cancers and to robustly confirm the unique features identified in our initial single-cell atlas.

## Processing of scRNA-seq data

Using Seurat v0.4.4.0, we processed the dataset by filtering out cells with less than 300 or more than 10,000 unique genes, mitochondrial gene expression above 20%, or total RNA counts exceeding 50,000. Specifically, cells were retained if they met the following criteria: nFeature_RNA > 300 and <10,000, percent.mt < 20, and nCount_RNA < 50,000. Doublet cells were identified using DoubletFinder v0.2.0.4 from CRAN [34], assigning each cell a doublet score, assigning a doublet score to each cell. Standard preprocessing included log-normalization and scaling to logarithmic gene expression values [12, 16, 35], followed by Principal Component Analysis (PCA) for dimensionality reduction. Cluster identification was based on the top 50 principal components, visualized using UMAP with the Seurat package. To enhance visualization capabilities, we incorporated the SCP package (version 0.5.1) [36]. We utilized the “epitools” R package for Ro/e calculations [37] and “ClusterGVis” and “org.Hs.eg.db” for marker gene analysis. Expression patterns were assessed with “AddModuleScore,” and pseudotime analysis was performed with “Monocle2” [38] and “slingshot” algorithms [39]. The “Scissor” R package [40] identified LAFs subtypes with significant prognostic associations in LUAD, and NicheNet analysis [41]was employed to predict potential ligands influencing transcriptomic changes and phenotypes.

To gain deeper insight into intercellular crosstalk within the TME, we performed ligand–receptor analysis using the CellChat R package (v1.1.1) [42]. Focusing on the “Secreted Signaling” category, we estimated communication probabilities and inferred signaling networks with the computeCommunProb, filterCommunication, and computeCommunProbPathway functions. Global cell–cell interaction networks were then summarized with aggregateNet, and selected signaling pathways were visualized as heatmaps using netVisual_heatmap.

## Constructing a prognostic signature using machine learning

To standardize data and enhance comparability, all datasets were normalized using Z-scores. The TCGA cohort served as the training set to explore the link between nLAF-related genes and LUAD survival, while other cohorts functioned as validation sets. The GSE31210, GSE50081, and GSE72094 cohorts were combined into a meta-LUAD cohort due to their limited sample sizes, and batch effects were corrected using the “ComBat” algorithm from the “SVA” package (Figure S1a&b).

In the initial step of model development, we identified 3486 upregulated genes in the TCGA-LUAD cohort and 1972 prognosis-associated risk genes through univariate Cox regression analysis. These genes were intersected with the top 200 nLAF-specific markers to refine the gene set for modeling, resulting in 54 candidate genes. Utilizing the Mime1 R package [43], we implemented 100 machine learning algorithm combinations, comprising 16 single algorithms and 84 paired combinations. Each algorithm was trained on the TCGA cohort, and the performance of the models was evaluated using the concordance index (C-index). Herein, Mime1 was used to implement a machine‑learning framework comprising 100 single and paired algorithm combinations based on the 54 nLAFrelated candidate genes. For single algorithms, the same method was used for both feature selection and prognostic model construction, whereas for paired combinations, the first algorithm performed gene selection and the second was used to build the prognostic model. To ensure robustness and avoid overfitting, K-fold cross-validation was performed within the training dataset. The models were then validated in the GEO-LUAD and meta-LUAD cohorts to assess their generalizability. Additionally, to further evaluate and clarify the performance and robustness of the model combinations selected based on C-index across different cohorts, we conducted a meta-analysis using the Mime1 R package. Mime1 also provides a comprehensive framework for integrating and comparing machine learning models, facilitating the assessment of model performance and robustness. To assess heterogeneity across different cohorts, we calculated the I^2^ statistic, which quantifies the percentage of total variation across studies that is due to heterogeneity rather than chance. An I^2^ value greater than 50% was considered to indicate significant heterogeneity. We performed sensitivity analysis by excluding one dataset at a time and recalculating the pooled hazard ratios (HRs) and confidence intervals (CIs). This process helped to identify any single dataset that might disproportionately influence the overall results. To evaluate publication bias, we used funnel plots and performed Egger’s regression test. A *p*-value less than 0.05 in Egger’s test was considered indicative of significant publication bias. The R package “metafor” (version 2.4.0) was used for calculating the I^2^ statistic and performing Egger’s regression test. The “survival” package (version 3.3–1) was used for Cox regression analysis.

Moreover, the nLAF Risk Score (nLRS) was calculated for each LUAD patient based on the optimal model, and patients were stratified into high- and low-nLRS groups using the median nLRS as the cutoff. Kaplan-Meier survival analysis was performed using the “survminer” R package to assess the prognostic significance of nLRS, with statistical significance set at *p* < 0.05. To benchmark the nLRS model, we compared its prognostic accuracy with 49 previously published LUAD-related signatures using the Mime1 R package. Additionally, prognostic nomogram models were constructed by integrating nLRS with clinical parameters (e.g., age, stage, and smoking history) in both the training and test LUAD cohorts. The predictive accuracy of these nomograms was evaluated using calibration curves and ROC curve analyses, implemented via the “rms”, “survival”, and “timeROC” R packages. This comprehensive approach ensured the reproducibility and robustness of the nLRS model.

## Immunological landscape and immunotherapy response analysis

Comprehensive data from the TCGA-LUAD cohort detailing TIME characteristics were used to explore the nLRS-associated immunological landscapes and immunotherapy responses. The Immuno-Oncology Biology Research (IOBR) R [44] package calculated enrichment scores for each sample, comparing tumor mutational burden (TMB), tumor neoantigen burden (TNB), CAFs, and myeloid-derived suppressor cells (MDSCs) between high- and low-nLRS groups. The immune exclusive signature scores, immune suppression signature scores, and immunotherapy biomarker scores between nLRS-high and low groups were also compared. Survival analysis was conducted to categorize patients according to their nLRS in the IMvigor210all immunotherapy-focused cohorts, with the assessment of immunotherapy response effectiveness determined by the survival delay observed in non-responders. This analysis was validated using three additional immunotherapy cohorts, including GSE135222, GSE78220, and GSE91061, to achieve a robust understanding of the relationship between nLRS and immunotherapy. Moreover, immunomodulatory molecules play a pivotal role in cancer immunotherapy, with numerous agonists and antagonists currently under evaluation in clinical oncology [45]. To further advance this field, it is essential to elucidate their expression patterns and regulatory mechanisms. In our study, we explored the expression of immunomodulatory molecules, somatic copy number alterations (SCNA), and the epigenetic control of gene expression. To further investigate the relationship between the target genes and immunotherapy outcomes, we employed the TIDE (Tumor Immune Dysfunction and Exclusion) analysis framework [46]. This approach allowed us to stratify immunotherapy data by treatment phase and perform Z-score normalization for each phase separately. This method enabled us to assess the relative expression levels of specific genes between responder and non-responder groups across different stages of treatment, thereby providing insights into the dynamic changes in gene expression associated with therapeutic response.

## Drug sensitivity analysis

We conducted a Gene Set Enrichment Analysis (GSEA) to identify differentially enriched pathways in tumor samples stratified by distinct nLRS, shedding light on the biological pathways potentially modulated by nLRS [47]. Utilizing the GDSC dataset, we predicted pharmacological responsiveness in LUAD patients, based on nLRS levels and key genes from our model. The chemotherapeutic susceptibility of LUAD patients was assessed by quantifying the half-maximal inhibitory concentration (IC50) values for a range of drugs, employing the “oncoPredict” R package [48].

## Spatial transcriptomics analysis

Utilizing the “Seurat” package (version 4.3.1), we undertook a comprehensive reanalysis of the six ST data relative to human pulmonary diseases, ensuring a rigorous and meticulous quality control process. This process was conducted in strict accordance with the methodologies previously detailed in the literature [17, 49]. Following this, we integrated the nLRS signature scores into the ST dataset’s metadata. This integration was achieved by employing the “AddModuleScore” function, utilizing the package’s default parameters to ensure consistency and reproducibility.

To assess the spatial distribution of the LAF subclusters identified from the single-cell cohort, we first merged the ST and the LAF scRNA-seq expression matrices and co-dimensioned them using the R package CellTrek [50]. Subsequently, we generated a sparse graph using a random forest model. This graph was then utilized to construct a spot-cell similarity matrix for the single cells, incorporating spatial coordinate information. This integrated approach enabled us to map the spatial distribution of the identified cell subclusters within the tissue context.

## Reverse transcription-quantitative polymerase chain reaction (RT-qPCR)

Total RNA was extracted from tumor and adjacent non-tumorous lung tissues using TRIzol reagent (Invitrogen) according to the manufacturer’s instructions. Subsequently, cDNA was synthesized using M‑MLV reverse transcriptase (XXX, Company, Country). RT‑qPCR was performed on a LightCycler® 480 System (Roche, Switzerland). Relative mRNA expression levels were calculated using the comparative Ct method with β‑actin as the internal control, and the ΔCt value was defined as: ΔCt = Ct(target gene)-Ct(β-actin). All data are presented as the mean ± standard error of the mean (SEM) from three independent experiments.

## Immunohistochemical (IHC) staining

Formalin-fixed paraffin-embedded lung tissue sections were deparaffinized with xylene and rehydrated through a graded alcohol series (100%, 85%, and 75% ethanol). Antigen retrieval was performed using citrate buffer (10 mM, pH 6.0) in a microwave (high power for 10 min, followed by medium-low power for 8 min). To quench endogenous peroxidase activity, slides were incubated in 3% hydrogen peroxide for 20 min at room temperature in the dark. After blocking with 3% BSA for 30 min at room temperature, tissue sections were incubated overnight at 4 °C with primary antibodies against HSP47 (1:300, 10,875–1-AP, Wuhan Sanying). After rinsing in PBS (pH 7.4), the sections were incubated with HRP-conjugated goat anti-rabbit secondary antibodies (1:200, GB23303, Servicebio) for 50 min at room temperature. Visualization was performed using freshly prepared diaminobenzidine (DAB) solution, and the reaction was stopped with tap water once brown staining was observed under a microscope. Hematoxylin counterstaining was performed to visualize nuclei, followed by dehydration through graded alcohols and xylene. Finally, the sections were mounted with neutral resin for microscopic examination.

## Western blot

Cells were lysed using RIPA buffer supplemented with protease and phosphatase inhibitors, and the protein concentration was quantified using a BCA assay. Equal amounts of protein were separated by SDS-PAGE and transferred onto a PVDF membrane. The membrane was then blocked with 5% non-fat milk, followed by incubation with specific primary antibodies overnight at 4 °C and a corresponding HRP-conjugated secondary antibody for 1 h at room temperature. Protein bands were finally visualized using an enhanced chemiluminescence (ECL) substrate, with β-Actin serving as a loading control.

## CCK-8 assay

Cells were seeded into a 96-well plate and incubated overnight. After treatment, 10 µL of CCK-8 solution was added to each well, followed by incubation at 37 °C for 1–4 h. The absorbance was measured at 450 nm, and cell viability was expressed as a percentage relative to the control group.

## Transwell assay

For the migration assay, cells in serum-free medium were seeded into the upper chamber of a Transwell insert, while complete medium with 10% FBS was added to the lower chamber as a chemoattractant. After 24–48 h of incubation, non-migrated cells on the upper surface were removed, and the migrated cells on the lower surface were fixed, stained with crystal violet, and counted. For the invasion assay, the same procedure was followed, except the upper chamber was pre-coated with Matrigel before cell seeding.

## Remark statement

The current study has been reported in line with the REMARK criteria [51].

## Statistical analysis

All statistical analyses were performed using R software, version 4.3.1. For assessing differences in continuous variables between two groups, we utilized the Student’s t-test, appropriate for normally distributed data. In cases where the data did not meet normality assumptions, the non-parametric Wilcoxon test was applied. For comparisons involving more than two groups, we employed a one-way ANOVA for parametric data, while the Kruskal-Wallis test was used for non-parametric data, allowing for a flexible approach to diverse data distributions. To explore relationships between continuous variables, Spearman’s correlation analysis was conducted.

Survival analysis was performed using Kaplan-Meier curves, and the log-rank test was employed to statistically compare these survival curves, determining differences in survival distributions across groups. To address the potential issue of multiple testing, especially in the context of multiple survival analyses, we applied the Bonferroni correction to adjust the significance threshold. This method controls the family-wise error rate by dividing the desired overall alpha level (typically 0.05) by the number of comparisons. Additionally, in some analyses involving a large number of simultaneous hypothesis tests, we controlled the false discovery rate (FDR) using the Benjamini-Hochberg procedure to adjust *p*-values. Throughout our analyses, a two-tailed *p*-value threshold of 0.05 was set to establish statistical significance, ensuring that our findings were both rigorous and reliable.

## Results

### Single-cell atlas reveals stage-specific microenvironmental dynamics in lung disease progression

**Fig. 1:**
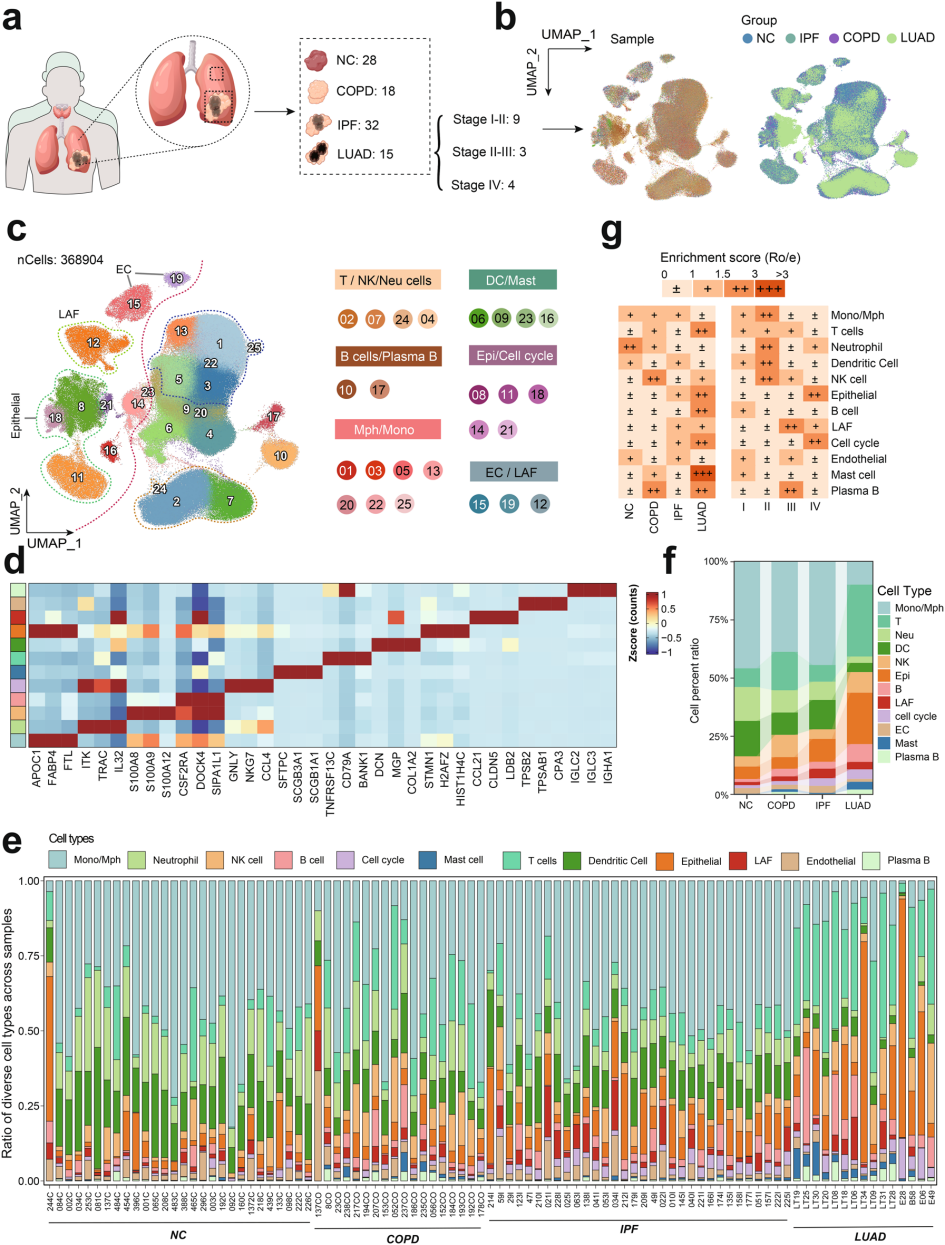
Single-cell RNA sequencing reveals cellular dynamics in lung disease progression. (**a**) UMAP visualization of single-cell RNA sequencing data from 93 lung disease samples, stratified by disease group. (**b**) Harmony-corrected UMAP of for the distribution of samples and groups, demonstrating the effective normalization of potential batch effects across the cohort. (**c**) UMAP representation of cells colored by various cell types. (**d**) The expression levels of representative signature genes across cell types. (e&f) Stacked barplot showing proportions of all cell types across samples and groups. Colors represent major cell populations. (**g**) Ro/e algorithm analysis indicating a specific enrichment of LAFs in IPF and LUAD tissues, with a more pronounced effect in advanced LUAD stages. LAFs, Lung associated fibroblasts; LUAD, Lung Adenocarcinoma

We conducted a comprehensive scRNA-seq analysis on a cohort of 93 samples, including 28 normal controls (NC), 18 chronic obstructive pulmonary disease (COPD), 32 idiopathic pulmonary fibrosis (IPF), and 16 lung adenocarcinoma (LUAD) samples (Fig. 1a). LUAD samples were stratified into stages I-II (9 samples), II-III (3 samples), and IV (4 samples) to capture the disease’s progression.

To address potential batch effects between samples, we applied the Harmony algorithm, effectively normalizing the data across the cohort (Fig. 1b). Following rigorous quality control measures, a dataset comprising 368,904 cells was subjected to further analysis. These cells were classified into 25 distinct clusters based on their gene expression profiles, which were annotated to represent various cell types, including neutrophils, B cells, plasma cells, mast cells, dendritic cells (DCs), natural killer (NK) cells, monocytes, lung-associated fibroblasts (LAFs), epithelial cells, endothelial cells, macrophages, T cells, and cell cycle (Figs. 1c&d, Figure S2a-c, Table S2). Of notice, we found LAFs exhibit increasing trend with the development of lung diseases (Fig. 1e&f). This trend was corroborated by the Ro/e algorithm, which indicated a specific enrichment of LAFs in the IPF and LUAD tissues, particularly in the advanced stages of LUAD (Fig. 1g). These findings suggest that LAFs may play a crucial role in the pathogenesis and progression of LUAD.

## Heterogeneity and functional specialization of LAFs during the progression of human lung disease

**Fig. 2:**
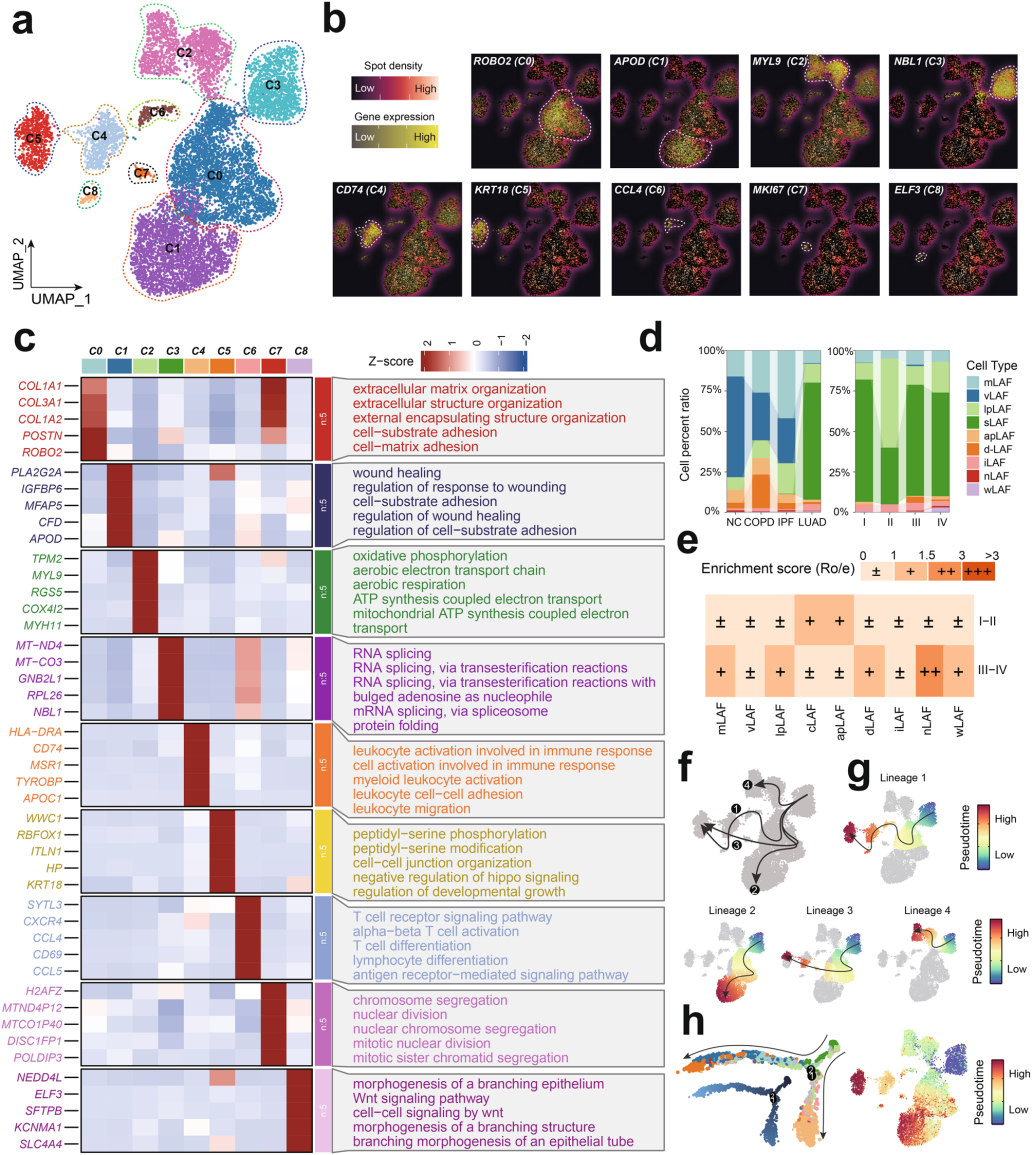
Heterogeneity and functional specialization of LAFs during the progression of human lung disease. (**a**) UMAP visualization of 10,550 LAFs, categorized into nine distinct subgroups through unsupervised clustering. (**b**) Heatmap depicting the expression of significant markers for LAFs and specific markers for clusters C0 to C8, illustrating the heterogeneity within the LAFs population. (**c**) The expression of top 5 significant markers and the presentation of top five enriched biological processes for each LAFs clusters, highlighting their functional specialization. (**d**) Stack plot showing the distribution of LAFs subtypes across the progression of lung disease stages, with a notable increase in the relative abundance of mLAFs, pLAFs, and nLAFs in tumor tissue compared to normal adjacent tissue. (**e**) Enrichment score (Ro/e) analysis, indicating a pronounced tissue enrichment of nLAFs in LUAD samples at advanced stages. (**f-h**) Pseudotemporal cell trajectories for various LAFs subtypes, constructed using the Slingshot algorithm and Monocle 2, revealing a bifurcated structure from a progenitor to a terminal effector state. LAFs, Lung associated fibroblasts; LUAD, Lung Adenocarcinoma; nLAFs, nuclear division LAFs

To investigate the role of LAFs in LUAD carcinogenesis and development, we re-analyzed 10,550 LAFs from our single-cell dataset, which were grouped into nine distinct clusters through unsupervised clustering (Fig. 2a). Markers for each cluster, from C0 to C8, along with the top five enriched biological processes, are detailed in Figure 2b&c and Table S3. We classified these clusters into specific LAF subtypes: myofibroblast-like (mLAFs), vascular (vLAFs), lipid-processing (lpLAFs), RNA splicing-related (sLAFs), antigen-presenting (apLAFs), development-related (dLAFs), immune (iLAFs), nuclear (nLAFs), and Wnt signaling pathway-related (wLAFs) LAFs. Markers used for each LAF subtype can be found in Table S4. Our analysis revealed an increased abundance of mLAFs, lpLAFs, and nLAFs in tumor tissue compared to normal tissue, particularly at advanced LUAD stages (Figs. 2d and 2e). We constructed pseudotemporal trajectories for these LAF subtypes using Slingshot and Monocle 2, which showed a bifurcated developmental path from progenitor to terminal effector states, with mLAFs and sLAFs initiating differentiation and nLAFs marking the end of this process (Fig. 2f–h).

**Fig. 3:**
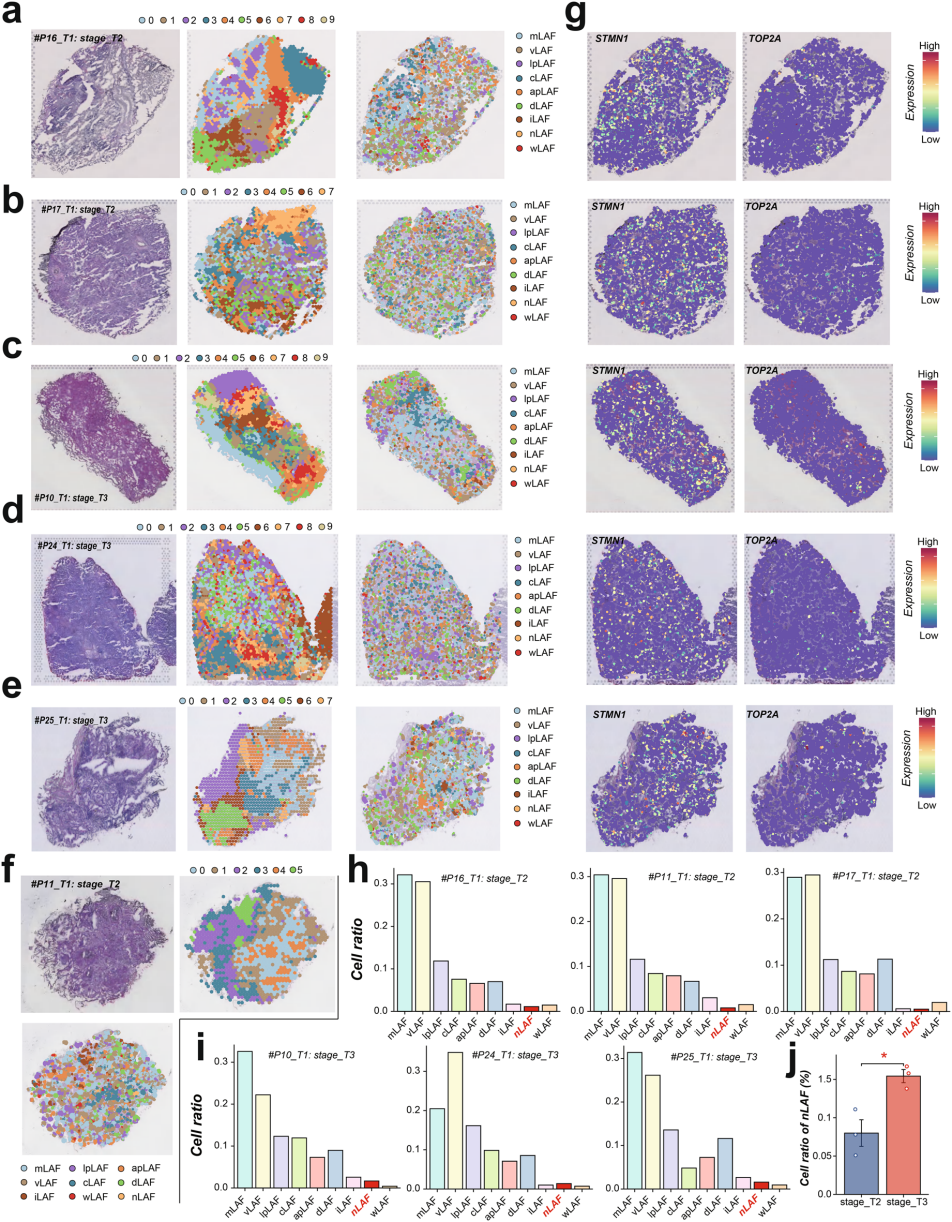
Spatial and temporal dynamics of LAF subtypes in LUAD. (**a-f**) Proportion of different LAF subtypes across various samples, analyzed using the CellTrek algorithm. (**g**) Expression levels of nLAF-associated genes (e.g., STMN1 and TOP2A) within different LAF subtypes. (**h-i**) Proportion of LAF subtypes (mLAF, vLAF, lpLAF, cLAF, apLAF, dLAF, iLAF, nLAF, wLAF) across all samples, highlighting the consistent predominance of mLAF and vLAF. (**j**) Comparison of nLAF proportions between T2 and T3 stages, revealing a significant increase in nLAFs from T2 to T3 stages, suggesting their potential role in LUAD progression

Notably, nLAFs exhibited the strongest enrichment in advanced stages of LUAD, suggesting a potential role in tumor progression and malignancy (Fig. 2e). To validate the role of nLAFs in the onset and progression of LUAD, we calculated the nLAF score in each patients from TCGA-LUAD cohorts using the ssGSEA algorithm, and noticed that higher nLAF score was associated with the carcinogenesis and development of LUAD (Figure S3a&b). To verify the proportion changes of nLAFs in LUAD tissues at different stages, we included ST data from three T2-stage and three T3-stage LUAD patients. Using the CellTrek algorithm, we analyzed the proportions of different LAF subtypes across these samples (Fig. 3a–f). The expression of nLAF-associated genes, such as STMN1 and TOP2A, within different LAF subtypes is shown in Fig. 3g. Our analysis revealed that mLAF and vLAF consistently accounted for the largest proportions across all samples, while nLAF and wLAF were present at much lower levels (Fig. 3h&i). Interestingly, the proportion of nLAFs was significantly lower in T2-stage patients compared to T3-stage patients, suggesting a potential association between nLAFs and LUAD progression (Fig. 3j). This observation prompted us to further investigate the characteristics of nLAFs and compare them with other fibroblast subtypes across different solid tumors.

## Comparison of nLafs with other fibroblast subtypes and validation in LUAD

**Fig. 4:**
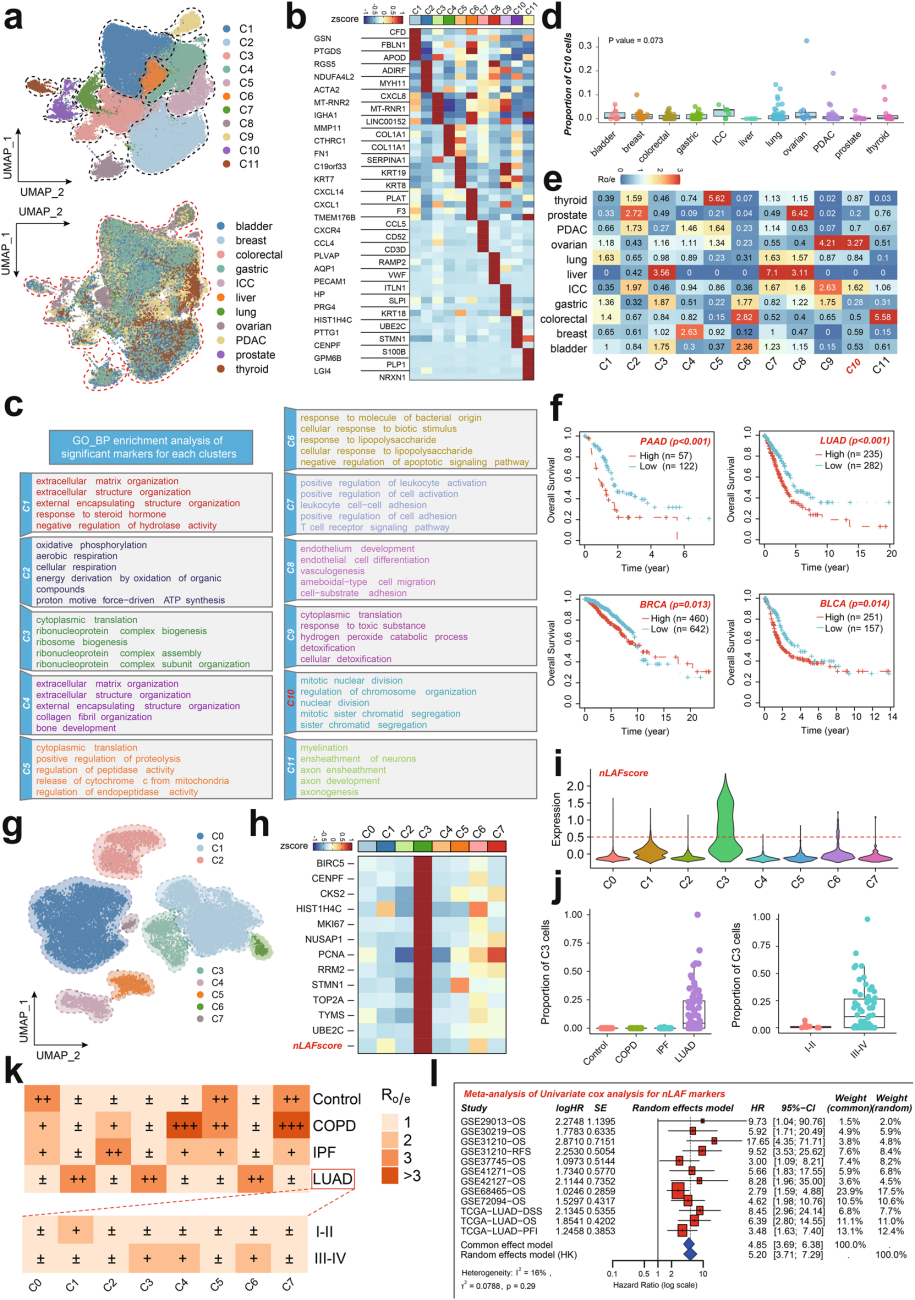
Pan-Cancer analysis and validation of nLAFs (**a**) UMAP visualization of 104,804 fibroblasts from 226 samples across 10 solid cancer types, clustered into 11 distinct subtypes. (**b**) Differential gene expression heatmap for the identified fibroblast clusters. (**c**) GO-BP enrichment analysis highlighting pathways enriched in cluster C10, similar to nLAFs, particularly cell proliferation-related pathways. (**d-e**) Proportion of C10 cells across different cancer types. (**f**) Association of nLAF scores with prognosis across various cancers, indicating poor prognosis in pancreatic, lung, bladder, and breast cancers. (**g**) UMAP visualization of 15,657 re-integrated lung-associated fibroblasts from an expanded LUAD cohort. (**h-i**) Identification of cluster C3 with high expression of nLAF-related markers and nLAF score, indicative of its proliferative nature. (**j**) Box plots showing the proportion of C3 cells in LUAD samples, higher in advanced stages. (**k**) Tissue enrichment preferences of C3 cells in LUAD. (**l**) Meta-analysis of survival hazard ratio of nLAFs across various LUAD cohorts. Meta-analysis of univariate Cox survival analysis results across multiple LUAD cohorts using the inverse variance method. The primary measurement indicator is the logarithm of the hazard ratio (HR). HR values are categorized into two groups: less than 1 (indicating a protective effect against cancer) and greater than 1 (indicating a pro-cancer effect). This categorization does not account for the underlying regulatory mechanisms associated with the studied genes. Statistical analysis and visualization were performed using the “Meta” package in R (version 4.3.2)

To further elucidate the characteristics of the identified nLAFs and compare them with previously described fibroblast subtypes in other solid tumors, we performed a comprehensive pan-cancer reanalysis using the GSE210347 dataset. In this analysis, we profiled 226 samples across 10 solid cancer types, identifying a total of 104,804 fibroblasts. These fibroblasts were subjected to 11 distinct clusters (Fig. 4a). The differential gene expression heatmap (Fig. 4b) and GO-BP enrichment analysis (Fig. 4c) revealed that cluster C10 exhibited gene expression signatures similar to nLAFs, with significant enrichment in cell proliferation-related pathways such as mitotic nuclear division and chromosome organization.

We further compared the proportion of C10 cells across different cancer types and found that this proliferative CAF subtype was relatively enriched in lung cancer, ovarian cancer, and intrahepatic cholangiocarcinoma (ICC) (Fig. 4d&e). Additionally, our pan-cancer analysis highlighted significant heterogeneity among different fibroblast subclusters, each with distinct pathway enrichment functions and tissue preferences. For instance, matrix-related CAFs (mCAFs, clusters C1 and C4) were enriched in bladder cancer, gastric cancer, and ovarian cancer, while immune-related CAFs (iCAFs, cluster C7) were enriched in liver cancer, lung cancer, ICC, and bladder cancer. Using the ssGSEA method, we calculated the nLAF score across various cancers and found that higher nLAF scores were associated with poor prognosis in pancreatic cancer, lung cancer, bladder cancer, and breast cancer (Fig. 4f), suggesting a role for nLAFs in promoting tumor malignancy. Importantly, while nLAFs share core proliferative pathways, their prevalence and functional roles appear to vary depending on the tumor microenvironment, reflecting both conserved and context-specific features of fibroblast biology.

To address the potential limitations of our initial cohort, which included only four stage IV LUAD samples, we incorporated an additional 42 stage III-IV LUAD patients into our analysis. This expanded cohort was integrated with our previous single-cell data to serve as a validation set for investigating the role of nLAFs in LUAD. As shown in Fig. 4g, a total of 15,657 re-integrated lung-associated fibroblasts were divided into eight subclusters. Intriguingly, we identified cluster C3 as the subcluster with the highest and most specific expression of nLAF-related markers and nLAF score (Fig. 4h&i), indicating its proliferative nature. Moreover, the proportion of C3 cells was higher in LUAD, particularly in advanced stages (Fig. 4j), and exhibited specific tissue enrichment preferences (Fig. 4k). Consistent results were observed in multiple LUAD datasets, where the nLAF score was significantly associated with overall survival (OS), disease-specific survival (DSS), and progression-free interval (PFI) in LUAD (*p* < 0.05) (Fig. 4l). The hazard ratios (HR) were greater than 1, indicating a detrimental effect, and the meta-analysis results were in line with these observations, showing no significant heterogeneity across different datasets and survival endpoints.

## Prognostic significance of nLafs in LUAD

**Fig. 5:**
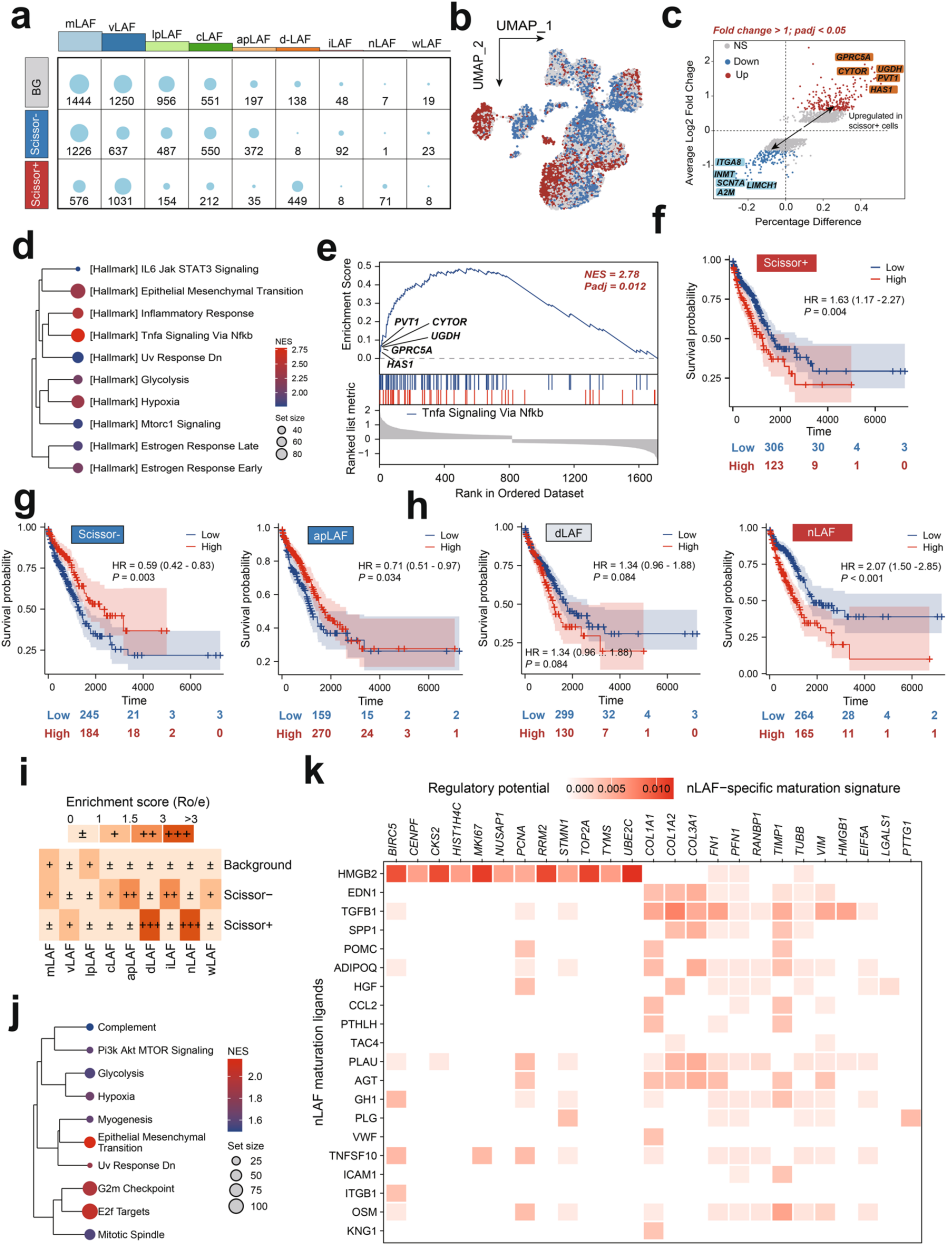
nLAFs associated with adverse prognosis in LUAD. (a&b) Distribution of Scissor-, Scissor+, and background (BG) cell numbers in each LAFsubtypes. (**c**) Volcano plot displaying the differentially expressed genes between scissor+ and scissor- LAFs. (**d-e**) Enrichment analysis of the upregulated genes in scissor+ LAFs, showing primary enrichment in pathways related to epithelial-mesenchymal transition (EMT), hypoxia, and tumor-related signaling pathways. (**f-h**) Kaplan-Meier survival curves for LUAD patients stratified by the enriched scores of various LAFs subtypes calculate by the ssGSEA based on their significant markers. (**i**) Heatmap showing tissue preferences of LAFs subtypes in each groups divided by Scissor algorithm, revealed by Ro/e. (**j**) Enrichment analysis of differential genes in nLAFs, revealing pathways similar to those upregulated in scissor+ LAFs. (**k**) Network plot from the NicheNet algorithm, identifying HMGB2 as a potential key ligand regulating the expression of nLAFs marker genes. LAFs, Lung associated fibroblasts; LUAD, Lung Adenocarcinoma; nLAFs, nuclear division LAFs

To determine the LAFs subtypes associated with LUAD prognosis, we utilized the scissor algorithm, which integrates comprehensive bulk RNA and scRNA-seq data to identify subpopulations closely linked to disease phenotypes. This approach allowed us to distinguish scissor+ cells within LAFs subpopulations that are correlated with poor LUAD prognosis. Our analysis revealed that the majority of dLAFs and nLAFs were classified as scissor+ (Fig. 5a, b).

We then conducted a differential expression analysis between scissor+ and scissor- LAFs, identifying 231 genes that were significantly upregulated in scissor+ LAFs cells (average log2 fold change > 0.58, adjusted *p*-value < 0.001, Fig. 5c, Table S5). Further enrichment analysis indicated that these upregulated genes were primarily enriched in pathways associated with epithelial-mesenchymal transition (EMT), hypoxia, and tumor-related signaling pathways, such as TNFα, with the top markers significantly enriched in the TNFα pathway (Fig. 5d&e). Higher scores of scissor+ and nLAFs were associated with poorer LUAD prognosis, while elevated scores of scissor- LAFs and apLAFs were related to better clinical outcomes (Fig. 5f–h). As expected, nLAFs was significantly enriched in the scissor+ cell subpopulation (Fig. 5i). Enrichment analysis of differential genes in nLAFs revealed pathways similar to those upregulated in scissor+ LAFs (Fig. 5j). To identify potential ligand-receptor pairs that regulate the existence and phenotypes of nLAFs, we utilized the NicheNet algorithm. Interestingly, we identified HMGB2 as a potential key ligand that regulates the expression of nLAFs marker genes, suggesting a critical role for HMGB2 in the regulation of nLAFs (Fig. 5k).

## Development and validation of nLRS prognostic models for LUAD using machine learning techniques

**Fig. 6:**
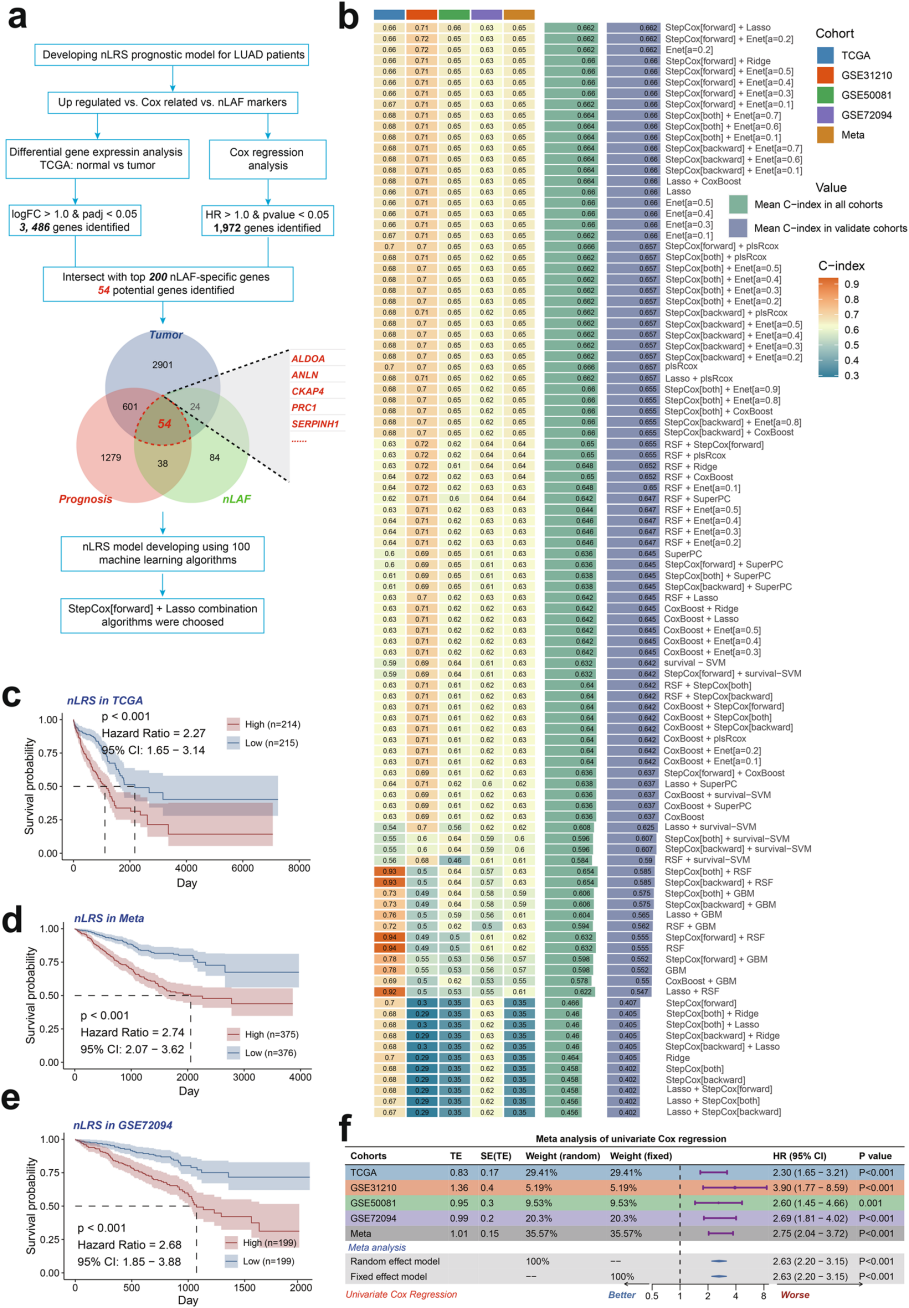
Development and validation of nLRS prognostic models for LUAD using machine learning techniques. (**a**) Flow diagram illustrate the process of gene selection and the steps involved in preparing the genes for machine learning analysis. (**b**) Through a comprehensive computational framework, a combination of 100 machine learning algorithms was generated. The C-index of each model was calculated through the TCGA-LUAD, GSE31210, GSE50081, GSE72094, and Meta-LUAD cohorts and sorted by the average C-index of the validation set, with the model combining StepCox and Lasso showing the highest predictive efficacy. (**c-e**) Kaplan-Meier survival curves for patients in the training and testing cohorts, categorized into high and low nLRS groups based on the median risk score. (**f**) Meta-analysis forest plot of univariate COX regression, confirming nLRS as a robust prognostic marker for patients with LUAD across the training and testing cohorts. LAFs, Lung associated fibroblasts; LUAD, Lung Adenocarcinoma; nLAFs, nuclear division LAFs; nLRS, nLAFs risk score

In our study, we identified 3486 upregulated genes and 1, 972 prognosis-related genes in LUAD using TCGA data and intersected them with top 200 nLAF markers to obtain 54 relevant genes (Fig. 6a, Table S6&7). We then applied a machine learning framework with 100 algorithms to construct a prognostic model, finding that a combination of StepCox and Lasso showed the highest predictive accuracy (Fig. 6b). This process identified five model genes, namely ALDOA, ANLN, CKAP4, PRC1, and SERPINH1, which showed significant prognostic value across all LUAD cohorts through Cox regression analysis (Figure S4a-e, Table S8&9). Based on the StepCox plus Lasso model, we calculated the nLRS in each LUAD cohort with the following formula: ALDOAexp × 0.110146 + ANLNexp × 0.23488 + CKAP4exp × 0.046603 + PRC1exp × 0.675478 + SERPINH1exp × 0.053045. Patients with high nLRS scores had a poorer prognosis (*p* < 0.001) (Fig. 6c–e), and a meta-analysis confirmed the nLRS as a strong prognostic factor for LUAD (Fig. 6f).

## Spatial transcriptomics reveals elevated nLRS levels in LUAD tissues

**Fig. 7:**
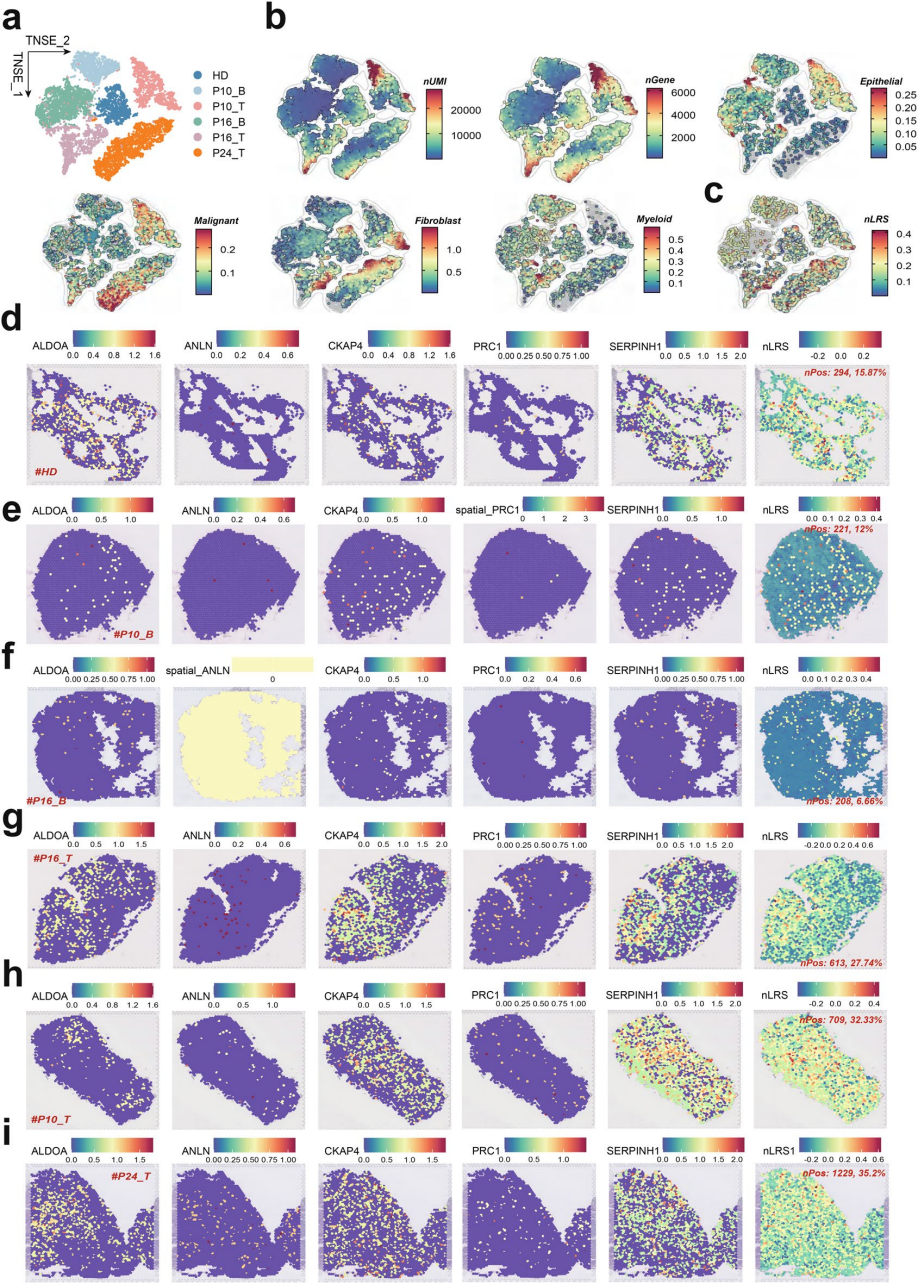
Spatial transcriptomics reveals elevated nLRS levels in LUAD tissues. (**a**) UMA plots showing spots from all sections, including one healthy lung tissue, two adjacent non-cancerous tissues, and three LUAD tissues, illustrating the spatial context of the samples. Color-coded according to samples. (**b**) Signature-based strategy to quantify the enrichment of various cellular components within each spot, highlighting the complexity of cellular constituents in each sampled point. (**c**) Spatial distribution maps of nLRS levels across the samples, demonstrating a significant increase in LUAD tissues compared to non-tumor control groups. (**d-i**) Graphs representing the expression of nLRS model genes and nLRS levels, as well as the proportion of nLRS-positive cells in each ST sample, with LUAD tissues showing a significant increase in both metrics. LAFs, Lung associated fibroblasts; LUAD, Lung Adenocarcinoma; nLAFs, nuclear division LAFs; nLRS, nLAFs risk score

We conducted ST analysis on diverse lung tissues, including healthy and LUAD samples, to map the nLRS and associated gene expression (Fig. 7a). Using a signature-based approach, we quantified cellular component enrichment in each spot, revealing cellular heterogeneity, particularly in fibroblast populations (Fig. 7b). Utilizing the “AddModuleScore”, we calculated the nLRS score spots with a nLRS score above zero were categorized as positive. Interestingly, we found the nLRS levels were significantly higher in LUAD tissues than in non-tumor controls, both in score and positive cell proportion (Fig.s 7c-i). These findings underscore the potential of nLRS as a spatial biomarker in LUAD, reflecting the intricate interplay between gene expression patterns and tumorigenesis.

## Comparative prognostic value of the nLRS and construction of a nomogram model

**Fig. 8:**
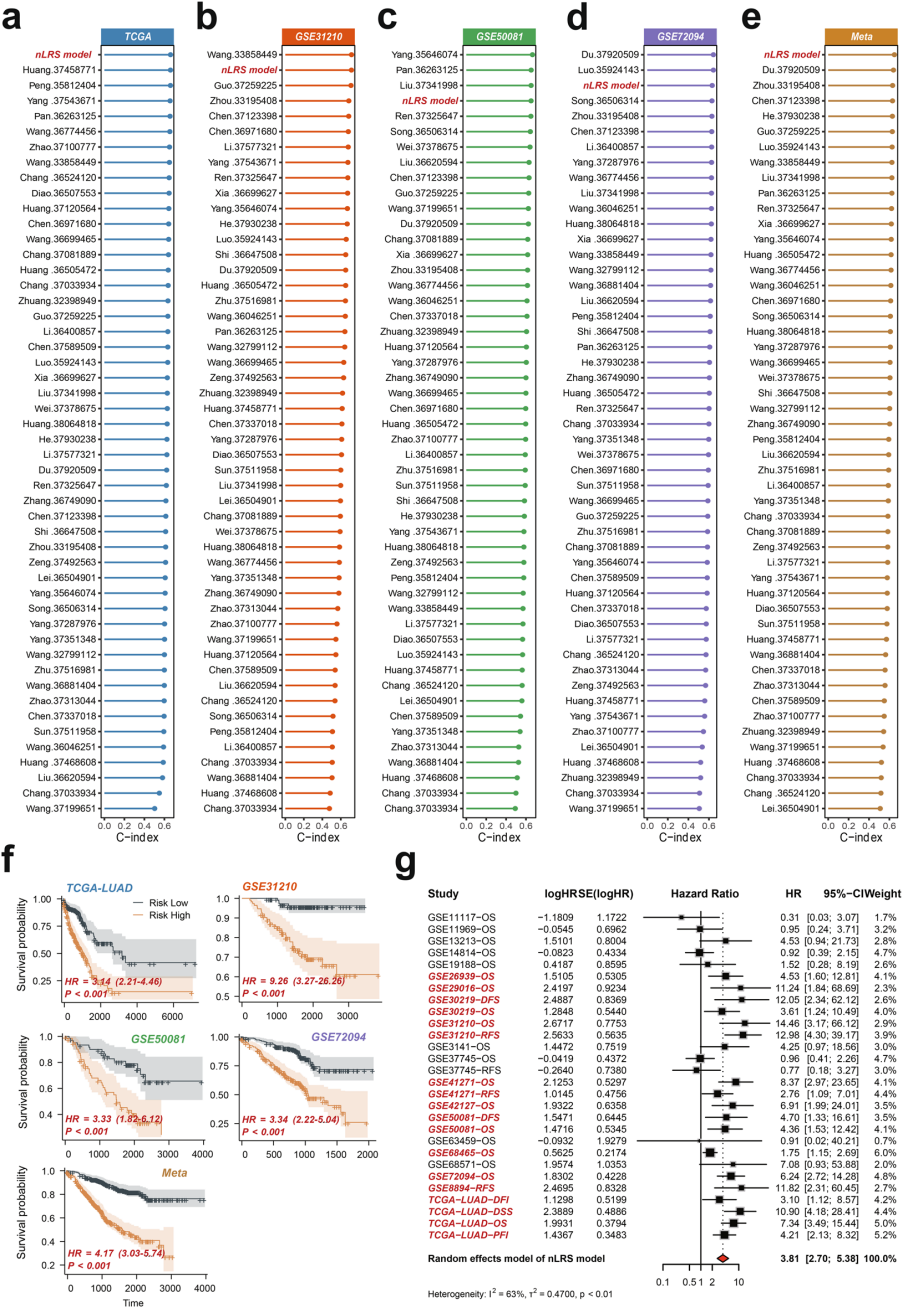
Comparative prognostic value of the nLRS and construction of a nomogram model. (**a-e**) C-index of nLRS model compared to 49 other established prognostic models in various LUAD datasets. (**f**) Kaplan-Meier curves highlighting the clinical relevance of the nomogram model, showing significant differences in survival probabilities between high and low risk groups. (**g**) Forest plot from a meta-analysis of univariate Cox survival analyses, confirming that the nLRS is a significant risk factor for LUAD prognosis. Meta-analysis of survival HR of nLRS across various LUAD cohorts. Meta-analysis of univariate Cox survival analysis results across multiple LUAD cohorts using the inverse variance method. The primary measurement indicator is the logarithm of the HR. Statistical analysis and visualization were performed using the “Meta” package in R (version 4.3.2). LAFs, Lung associated fibroblasts; LUAD, Lung Adenocarcinoma; nLAFs, nuclear division LAFs; nLRS, nLAFs risk score

To assess the predictive power of our nLRS model for LUAD prognosis, we systematically reviewed the literature and compared our model’s c-index with 49 established models. Our nLRS demonstrated superior performance across various datasets (Fig. 8a–e, Table S10). Before clinical implementation, we confirmed the nLRS’s predictive and prognostic significance by quantifying its levels in 2719 LUAD patients from 20 cohorts, which validated its prognostic value (Table S1, Fig. 8f). A meta-analysis of univariate Cox survival analyses confirmed the nLRS as a significant risk factor for LUAD prognosis (*HR* = 3.81, *p* < 0.001, Fig. 8g).

We then integrated the nLRS with clinical parameters in multivariate Cox and stepwise regression analyses to construct nomogram models, enhancing the model’s accuracy (Figure S5a, Table S11-15). Validation through calibration curve and ROC analyses showed excellent performance (Figure S5b&c), and Kaplan-Meier curves underscored the model’s clinical applicability (Fig. 8g). To further explore the incremental utility of the nLRS model in clinical decision-making compared with other models incorporating readily available biomarkers, we conducted a comprehensive analysis. Specifically, we compared the nLRS model with models based on well-established biomarkers such as CEACAM5/6, MUC16, and KRT19, which are commonly used in clinical settings for lung cancer diagnosis and prognosis [52–54].

Interestingly, our results showed that the nLRS model outperformed these traditional biomarkers in both LUAD diagnosis and prognosis (Figure S3c, f). This suggests that the nLRS model may provide more accurate and reliable information for clinical decision-making compared to the currently used biomarkers. To further assess the incremental utility of the nLRS model, we combined it with the above biomarkers to construct nomogram models for clinical decision support (Figure S3d, g). The results indicated that the combined model showed incremental value in LUAD diagnosis (Figure S3e), suggesting that incorporating the nLRS model could enhance the diagnostic accuracy beyond what is achievable with the traditional biomarkers alone. However, no significant incremental value was observed in prognosis (Figure S3h&i), indicating that while the nLRS model improves diagnostic performance, its addition does not significantly enhance prognostic predictions when combined with existing biomarkers. However, when combined with other clinical parameters such as stage, smoking status, and age, demonstrated significant incremental value in prognostic assessments (Figure S5a-c). This suggests that while the nLRS model improves diagnostic performance, its true potential in prognostic applications is realized when integrated with additional clinical parameters. The combination of the nLRS model and these clinical parameters provides a more comprehensive and accurate assessment of patient outcomes, highlighting the importance of a multifaceted approach in clinical decision-making.

## Distinct immune landscapes linked to the nLafs risk score in LUAD

**Fig. 9:**
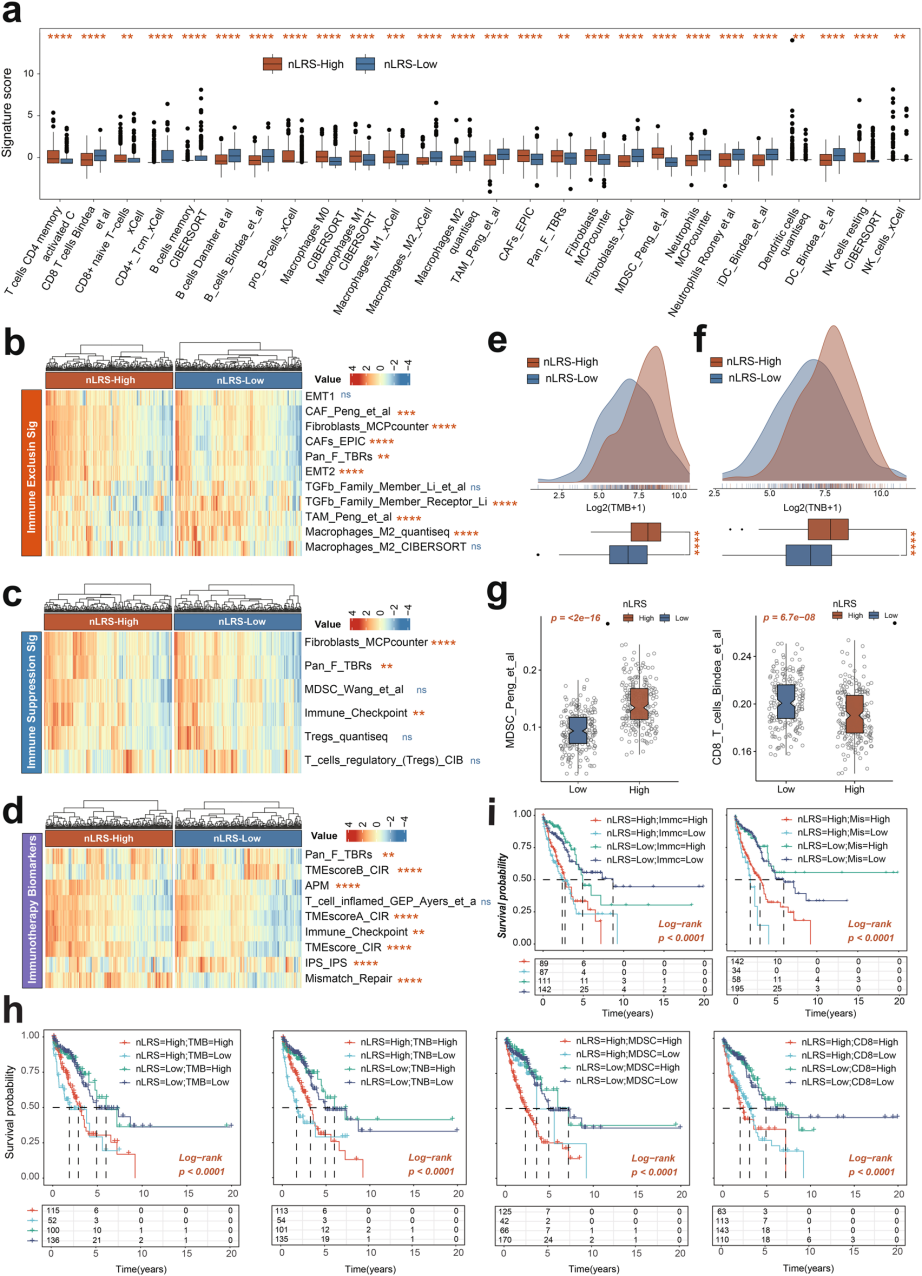
Distinct immune landscapes linked to the nLAFs risk score in LUAD. (**a**) Box plots displaying the immune cell infiltration levels in the nLRS-High and nLRS-Low groups. (**b-d**) Heatmaps illustrating the distribution of immune exclusion immune suppression, and immunotherapy-related signatures between high- and low-nLRS patients in LUAD. (e&f) The distribution of TMB and TNB between high- and low-nLRS patients in LUAD. (**g**) The distribution of MDSC and CD8+ T cells between high- and low-nLRS patients in LUAD. (**h**) Survival analysis combined nLRS with TMB, TNB, MDSC, and CD8+ T cells in LUAD patients. (**i**) Survival analysis combined nLRS with Immune_Checkpoint and Mismatch_Repair LUAD patients. LAFs, Lung associated fibroblasts; LUAD, Lung Adenocarcinoma; nLAFs, nuclear division LAFs; nLRS, nLAFs risk score; MDSCs, myeloid-derived suppressor cells; TMB, Tumor mutational burden; TNB, Tumor neoantigen burden; Immc: Immune_Checkpoint, MMR: Mismatch_Repair. *p < 0.05, **p < 0.01, ***p < 0.001, ns = not significant

To investigate the immune landscape associated with nLAFs in LUAD, we utilized the “IOBR” R package for a thorough analysis of immune cell infiltration within the tumor microenvironment (TME). Notably, the nLRS-high group showed elevated levels of CD8+ naive T-cells, Macrophages M1, MDSCs, and Fibroblasts MCP counter, indicating an immune suppressive phenotype (Fig. 9a). This group also had increased Fibroblasts_MCPcounter and MDSC_Wang_et_al scores, suggesting enhanced immune evasion (Fig. 9b&c). Consistently, immune-checkpoint therapy biomarkers were upregulated in the nLRS-high group, potentially contributing to immunosuppression (Fig.s 9d). Additionally, the nLRS-high group had higher TMB and TNB, suggesting a more complex immune interaction (Fig.s 9e&f). Survival analysis revealed that nLRS scores, in conjunction with TMB, TNB, CD8+ T cell infiltration, and MDSC infiltration, could effectively stratify patient prognosis, with lower nLRS scores and MDSC infiltration associated with better LUAD survival outcomes (Fig. 9h). Immune checkpoint expression and mismatch repair (MMR) levels are well-established predictors of patient response to immune checkpoint blockade (ICB) therapy [55, 56]. Our analysis revealed that nLRS-high patients exhibit significantly elevated levels of immune checkpoint markers and MMR-related genes, suggesting that nLRS may play a critical role in shaping the tumor microenvironment and influencing immunotherapy responses. To further explore potential combination strategies, we stratified patients based on their nLRS levels and MMR status. Interestingly, patients with nLRS-low and MMR-high profiles demonstrated the most favorable clinical outcomes, including significantly prolonged survival (Fig. 9i). This observation highlights the potential therapeutic benefit of combining nLRS-low status with ICB therapy, particularly in patients with high MMR activity.

**nLRS guide immunotherapeutic outcomes and personalized treatment Strategies in LUAD**

**Fig. 10:**
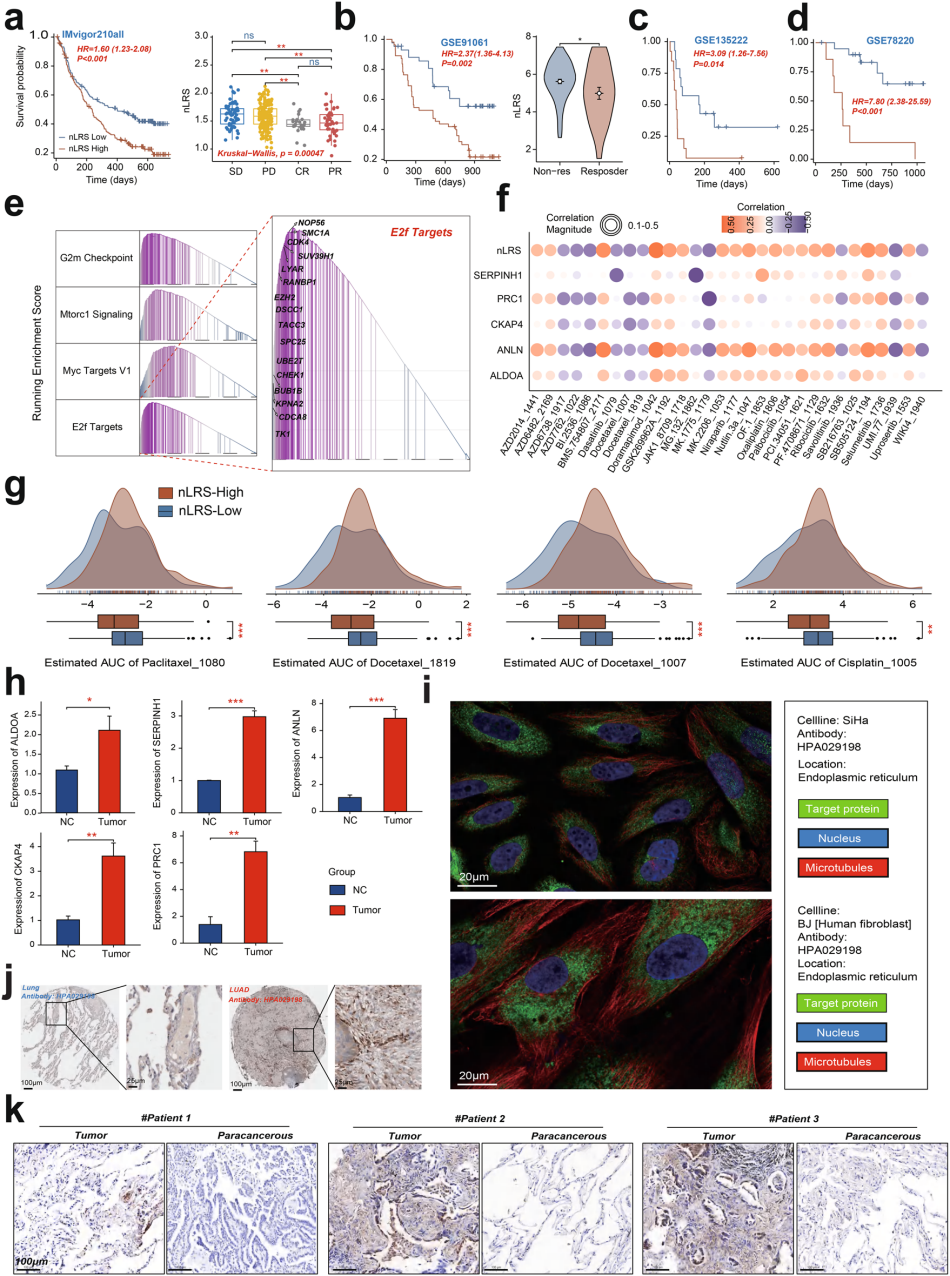
nLRS guide immunotherapeutic outcomes and personalized treatment strategies in LUAD. (**a**) Left panel shows the Kaplan-Meier survival curves for patients with low and high nLRS from the IMvigor210 cohort, and the right panel depicting the differences of nLRS levels among LUAD patient with various responses to immunotherapy, with statistical significance indicated. (**b**) Left panel shows the Kaplan-Meier survival curves for patients with low and high nLRS from the GSE91061 cohort, and the right panel depicting the differences of nLRS levels among LUAD patient with various responses to immunotherapy, with statistical significance indicated. (c&d) Kaplan-Meier survival curves for patients with low and high nLRS from the GSE135222 and GSE78220 cohorts. (**e**) Enrichment plots of EMT and cell cycle-related pathways in high nLRS groups, suggesting a more aggressive disease phenotype due to enhanced immune evasion mechanisms. (**f-g**) Dot and scatter plots illustrating the negative correlation between nLRS and sensitivity to chemotherapeutic agents and targeted therapies, such as cisplatin, docetaxel, paclitaxel, and gefitinib, indicating potential treatment options for high nLRS patients. (**h**) Validation of the differential expression of five model genes through RT-qPCR analysis through clinical LUAD sample. (**i**) Immunohistochemistry images from the HPA database showing the subcellular distribution and protein expression levels of SERPINH1. (**j**) Relative protein levels of SERPINH1 in LUAD tissue compared to a normal tissue, as determined by the HPA database. (**k**) Immunohistochemical analyses of SERPINH1 in clinical samples. LAFs, Lung associated fibroblasts; LUAD, Lung Adenocarcinoma; nLAFs, nuclear division LAFs; nLRS, nLAFs risk score; *p < 0.05, **p < 0.01, ***p < 0.001

To determine the impact of nLRS on LUAD immunotherapy outcomes, we analyzed the IMvigor210 cohort, finding that patients with low nLRS had significantly better survival rates (*HR* = 1.60, *p* < 0.001; Fig. 10a). LUAD patients respond favorably to immunotherapy had lower nLRS scores than non-responders, indicating a potential predictive value for immunotherapy response. This trend was consistent across three additional independent cohorts (Fig. 10b–d). GSEA in high nLRS groups revealed enrichment in EMT and cell cycle pathways, suggesting a more aggressive disease phenotype (Fig. 10e). Notably, the immunotherapy cohorts analyzed—including IMvigor210 (urothelial carcinoma), GSE135222 (melanoma), GSE78220 (melanoma), and GSE91061 (melanoma)—are not LUAD-specific. Consequently, these findings represent exploratory pan-cancer associations that warrant validation in prospective LUAD immunotherapy trials. We present these data as hypothesis-generating evidence, and their applicability to LUAD remains to be determined.

Interestingly, we found an inverse correlation between nLRS and sensitivity to chemotherapeutic agents and targeted therapies based on data from the GDSC database, including cisplatin, docetaxel, paclitaxel, and gefitinib (Fig. 10f&g, Table S16), implying potential personalized treatment options. To substantiate the expression patterns of our identified model genes, we performed RT-qPCR validation studies on clinical LUAD samples, encompassing both adjacent and cancerous tissues (Table S17). The expression levels of the five nLRS model genes were consistently higher in LUAD tissues, aligning with our bioinformatics findings and thereby validating the robustness of our study (Fig. 10h).

In light of the highest HR value attributed to SERPINH1 in our univariate Cox regression analysis, we proceeded to investigate its subcellular distribution and protein expression levels using the HPA database. SERPINH1 was predominantly found in the endoplasmic reticulum, with a significant upregulation in LUAD tissues compared to normal controls (Fig.s 10i, j). Subsequent IHC staining substantiated the elevated expression of SERPINH1 in tumor samples, as shown in Fig. 10k. Moreover, a higher SERPINH1 protein level was observed in LUAD tissues compared to normal tissues, as evidenced by data from the CPTAC-LUAD and LUAD_Academia datasets (Figure S6a & b). This underscores the potential of SERPINH1 as a critical biomarker for identifying high-risk populations in early lung cancer screening.

Given the pivotal role of immunomodulatory molecules in cancer immunotherapy [45], with numerous agonists and antagonists under clinical evaluation, understanding their expression patterns and regulatory mechanisms in the context of SERPINH1 is essential. In the SERPINH1_high group, immune-related genes were broadly upregulated (Figure S6c). Additionally, our investigation into somatic copy number alterations (SCNA) and epigenetic regulation revealed that SERPINH1 expression was closely linked to immune infiltration and genomic instability, as reflected by elevated immunogenicity and DNA damage scores (Figure S6d). These findings suggest that SERPINH1 may actively shape the tumor microenvironment through its impact on immune responses and genomic integrity. Consistent with these observations, data from pan-cancer immunotherapy cohorts [46] showed that high SERPINH1 expression was correlated with reduced sensitivity to immunotherapy and poorer clinical outcomes (Figure S6e & f). These results highlight the dual role of SERPINH1, both in driving tumor aggressiveness and modulating immune responses, thereby positioning it as a promising therapeutic target and prognostic biomarker for LUAD.

**nLAF-mediated recruitment of Mph_SPP1 subsets via COL1A1–CD44 Signaling**

**Fig. 11:**
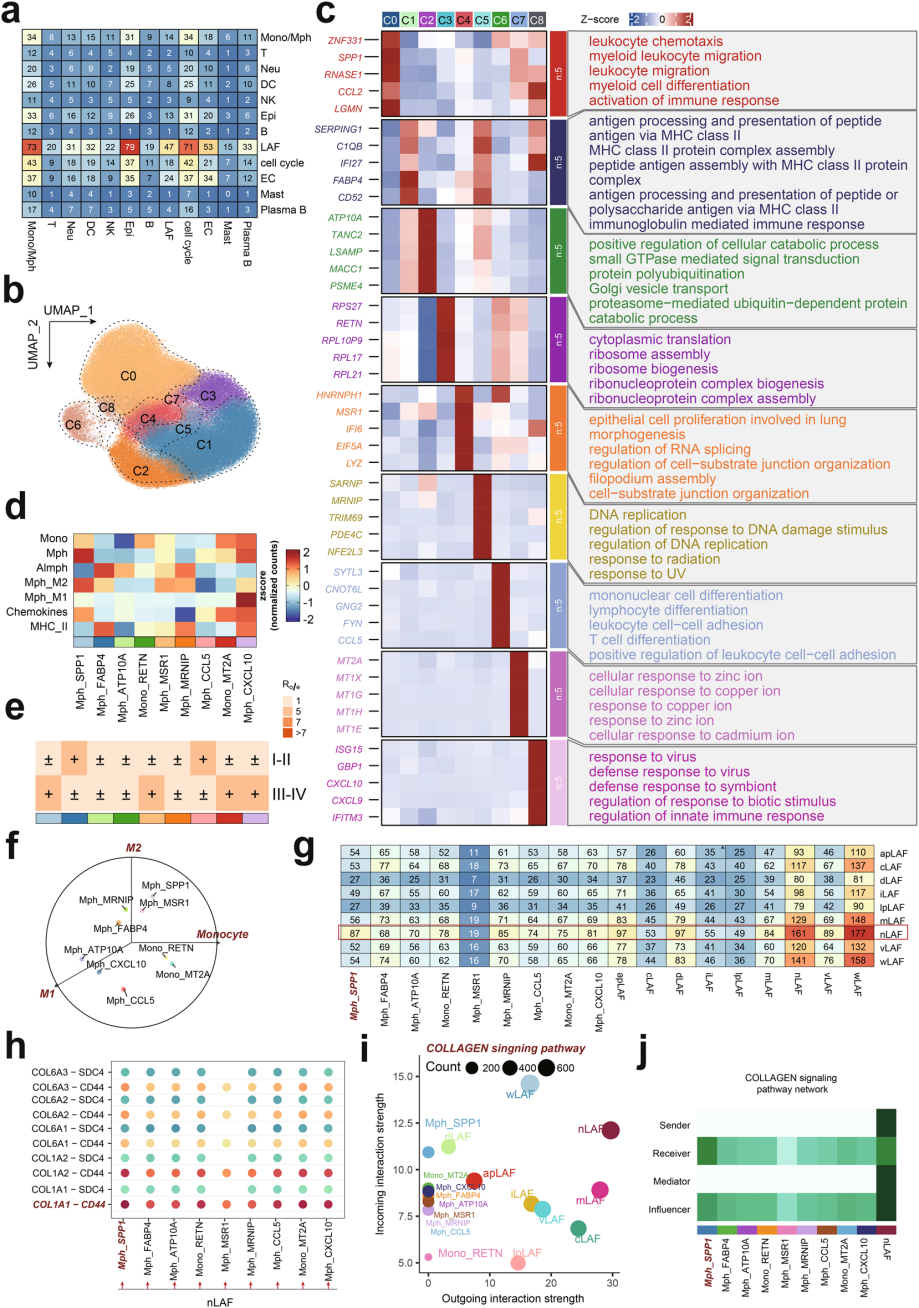
Cell-cell communication between nLAFs and monocyte/macrophage subpopulations in the lung immune microenvironment. (**a**) Heatmap showing the number of inferred ligand-receptor interactions among major cell types. Fibroblasts exhibit the highest interaction frequency with Monocyte/macrophage (Mono/Mph) populations. (**b**) UMAP visualization of 142,393 Mono/Mph cells, segregated into nine transcriptionally distinct subclusters (C0–C8). (**c**) Heatmap of representative marker genes for each Mono/Mph subcluster and their associated Gene Ontology (GO) terms, highlighting key functional programs. (**d**) Heatmap of pathway enrichment scores across Mono/Mph subclusters used to annotate subcluster identities. (**e**) Enrichment score (Ro/e) analysis, showing preferential enrichment of Mph_SPP1, Mph_MSR1, Mph_CXCL10, and Mono_MT2A in advanced disease. (**f**) Schematic summarizing functional polarization of Mono/Mph subclusters, highlighting M2-like macrophage phenotypes in Mph_SPP1 and Mph_MSR1. (**g**) Heatmap of the number of inferred ligand–receptor interactions between LAF subtypes and Mono/Mph subclusters, showing that nLafs have the strongest interaction with the Mph_SPP1 subcluster. (**h**) Dot plot of collagen-related ligand-receptor pairs between nLafs and Mono/Mph subclusters, with a prominent COL1A1–CD44 axis between nLafs and Mph_SPP1. (**i**) Scatter plot of outgoing versus incoming interaction strength for LAF and Mono/Mph populations in the COLLAGEN signaling pathway, indicating that nLAFs act mainly as signal senders, whereas Mph_SPP1 are major signal receivers. (**j**) Heatmap summarizing sender, receiver, mediator, and influencer roles of LAF and Mono/Mph populations within the COLLAGEN signaling network

To investigate whether nLAFs directly modulate immune cell recruitment or checkpoint expression, we first characterized cell-cell communication within the immune microenvironment during the fundamental progression of lung disease. This analysis revealed that fibroblasts, acting as signaling senders, exhibited the highest number of interactions with Monocyte/macrophage (Mono/Mph) populations among all immune cell types (Fig. 11a). We next extracted 142,393 Mono/Mph cells from our cohort and classified them into nine transcriptionally distinct subclusters (Fig. 11b). The marker genes and enriched functional pathways for each subcluster are shown in (Fig. 11c). Based on the expression patterns of subcluster-specific genes and their pathway enrichment scores (Fig. 11d), we annotated these populations and found that three macrophage subclusters (Mph_SPP1, Mph_MSR1, Mph_CXCL10) and one monocyte subcluster (Mono_MT2A) were significantly enriched in patients with more advanced-stage lung cancer (Fig. 11e). Notably, Mph_SPP1 and Mph_MSR1 displayed pronounced M2-like signatures, suggesting that these M2-polarized macrophage subsets may promote lung cancer progression, potentially by secreting anti-inflammatory mediators or facilitating angiogenesis (Fig. 11f).

To determine whether nLAFs directly participate in recruiting these poor-prognosis macrophage populations, we further examined intercellular communication between nLAFs and Mph subclusters. We observed that nLAFs exhibited the highest number of interactions with the Mph_SPP1 subcluster (Fig. 11g), with particularly strong signaling through the COL1A1–CD44 ligand-receptor axis (Fig. 11h). Interestingly, nLAFs functioned predominantly as signal senders, whereas Mph_SPP1 represented the major signal-receiving macrophage population (Fig. 11i&j). Correlation analysis suggests a potential association between nLAFs and Mph_SPP1 cells through COL1A1–CD44 expression patterns. However, whether this represents a direct signaling mechanism or a bystander association requires functional validation through ligand-receptor blocking experiments or genetic perturbation studies.

In summary, these results suggest that nLAFs may foster an immunosuppressive microenvironment in LUAD, at least in part by recruiting Mph_SPP1 via COL1A1–CD44 signaling. This mechanism provides a plausible cellular basis for the association between nLRS and adverse prognosis in LUAD and highlights SERPINH1 as a key mediator linking fibroblast activity to immune dysregulation and tumor progression. Further mechanistic studies are warranted to delineate the SERPINH1‑dependent pathways involved and to assess the feasibility of targeting SERPINH1 as a therapeutic strategy in LUAD.

## SERPINH1 is upregulated and promotes malignant progression in NSCLC

**Fig. 12:**
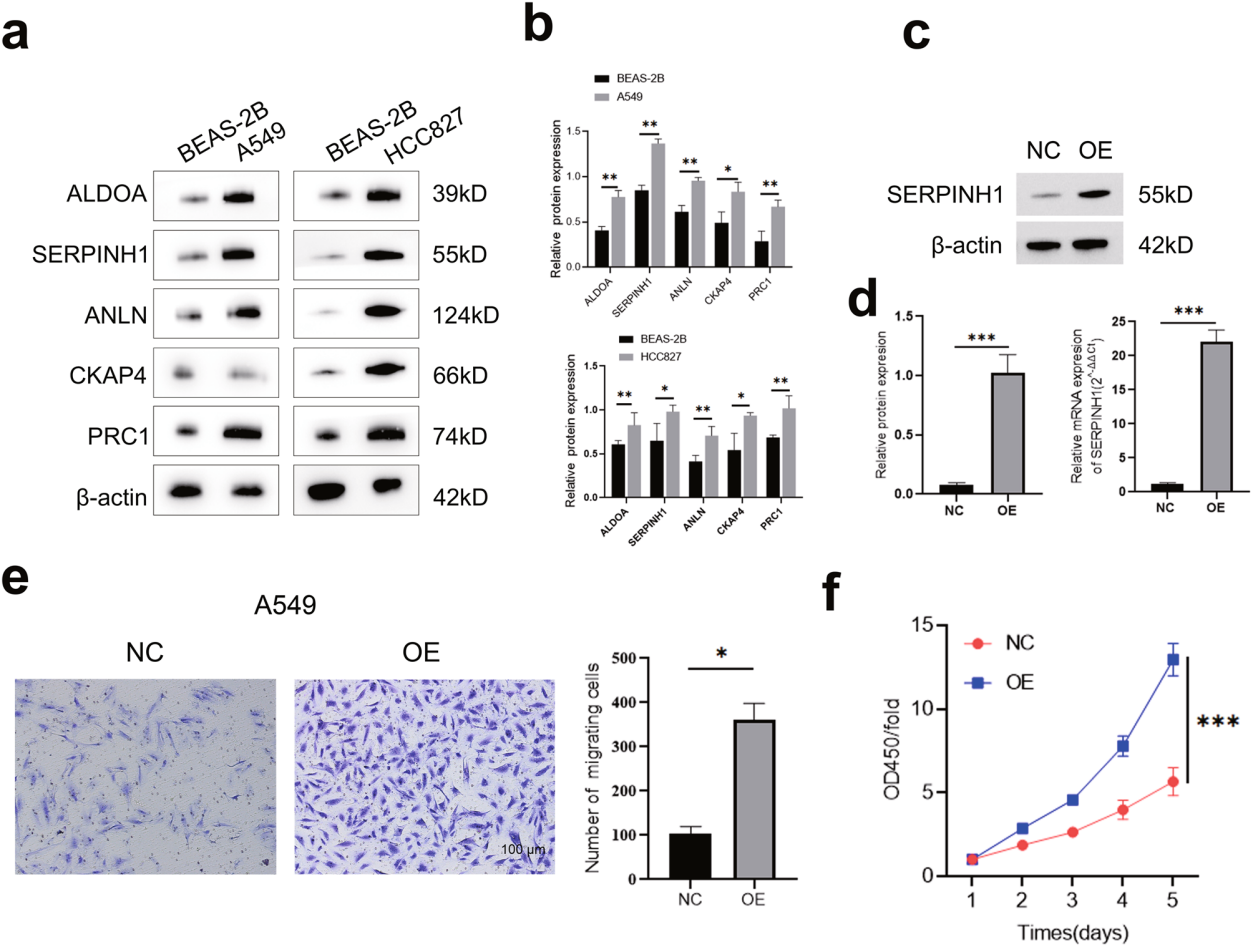
SERPINH1 is upregulated in NSCLC cells and promotes cell proliferation and migration. (**a**) Western blot analysis of ALDOA, SERPINH1, ANLN, CKAP4, and PRC1 protein expression in normal human bronchial epithelial cells (BEAS-2B) and NSCLC cell lines (A549 and HCC827). All five proteins showed significantly elevated expression levels in both A549 and HCC827 cells compared to BEAS-2B cells. Data are presented as mean ± SD of three independent experiments (*p < 0.05, **p < 0.01, ***p < 0.001). (**b**) Quantification of relative protein expression levels of the five genes in BEAS-2B versus A549 (upper) and BEAS-2B versus HCC827 (lower). Data are presented as mean ± SD of three independent experiments (*p < 0.05, **p < 0.01, ***p < 0.001). (**c**) Western blot validation of SERPINH1 overexpression in A549 cells stably transduced with SERPINH1 (OE) or negative control (NC) vectors. β-actin served as a loading control. (**d**) Quantification of SERPINH1 protein (left) and mRNA (right) expression in NC and SERPINH1-OE A549 cells. Data are shown as mean ± SD (***p < 0.001). (**e**) Transwell migration assay demonstrating that overexpression of SERPINH1 in A549 cells significantly enhanced cell migration capacity compared to control cells. Representative images (left) and quantitative analysis (right) of invaded cells are shown. Data are presented as mean ± SD of three independent experiments (*p < 0.05, **p < 0.01, ***p < 0.001). Scale bar: 100 μm. (**f**) Cell counting kit-8 (CCK-8) assay showing that SERPINH1 overexpression significantly promoted the proliferation of A549 cells compared to control cells at indicated time points. Data are presented as mean ± SD of three independent experiments (*p < 0.05, **p < 0.01, ***p < 0.001)

To identify potential oncogenes involved in NSCLC pathogenesis, we first screened the protein expression levels of five candidate genes (ALDOA, SERPINH1, ANLN, CKAP4, and PRC1) across different cell lines. Western blot analysis revealed that all five proteins were expressed at low levels in the normal human bronchial epithelial cell line BEAS-2B. In stark contrast, their expression was markedly elevated in two representative NSCLC cell lines, A549 and HCC827 (Fig. 12a). The concurrent upregulation at protein levels strongly suggests that these genes are potentially key drivers in NSCLC tumorigenesis and were selected for further functional investigation. Given its significant overexpression in NSCLC cells, we focused on SERPINH1 to elucidate its functional role (Fig. 12b). We established stable SERPINH1-overexpressing (OE) A549 cell lines to assess its impact on malignant behaviors (Fig. 12c&d). We initially evaluated the effect of SERPINH1 on cell proliferation using the CCK-8 assay. The results demonstrated that cells overexpressing SERPINH1 exhibited a significantly higher proliferation rate compared to the control cells over the course of 96 hours, indicating that SERPINH1 promotes the growth of lung cancer cells (Fig. 12e). Next, we investigated whether SERPINH1 influences metastatic potential, a critical aspect of cancer malignancy. Using a Transwell invasion assay, we found that the number of cells invading through the Matrigel-coated membrane was dramatically increased in the SERPINH1-OE group relative to the control group (Fig. 12f). This finding provides direct evidence that SERPINH1 enhances the invasive capacity of A549 cells.

## Discussion

In recent years, multi-omics analyses have emerged as a powerful tool in cancer research, providing unprecedented insights into the complex molecular landscapes of tumors [32, 33, 35, 49, 57]. By integrating diverse types of omics data, researchers can uncover the intricate interplay between different cellular components and their roles in disease progression. In this study, we have employed a multi-omics strategy, integrating bulk RNA, scRNA, and st-RNA sequencing data, encompassing 2719 LUAD patients, 368,904 single cells and 202,749 spatial spots to dissect the cellular heterogeneity throughout the progression of pulmonary diseases and the specific roles these play within the TME of LUAD. Our analysis has shed light on the pivotal role of LAFs in the development and progression of pulmonary diseases, providing a comprehensive view of LAFs in lung pathologies. Notably, we have found that LAFs are associated with more severe pulmonary diseases such as IPF and the progression of LUAD. Utilizing the scissor algorithm, we identified a subset of nLAFs significantly correlated with lung cancer patient prognosis and constructed the nLRS prognostic model, which includes five key genes: ALDOA, ANLN, CKAP4, PRC1, and SERPINH1. This model demonstrated superior predictive performance across multiple datasets, outperforming 49 previously published models. Furthermore, we observed that patients with high nLRS exhibit resistance to immunotherapy yet show increased sensitivity to chemotherapeutic and targeted agents.

Recent advances in single-cell and spatial transcriptomics have unveiled extensive heterogeneity among CAFs, with distinct subtypes implicated in tumor progression, immune modulation, and therapy resistance across cancers [12, 15–18]. For instance, mCAFs and immune-related CAFs have been linked to poor prognosis in pancreatic and breast cancers, respectively [20, 58]. Additionally, the significant heterogeneity within CAFs limits the clinical efficacy of direct targeting [15, 17]. The distribution and functional differences of distinct CAF subsets within the TME add further complexity to targeted therapies. Studies have shown that CAFs play a complex role in lung cancer progression, promoting tumor growth and metastasis while potentially inhibiting tumor progression in certain contexts [59–61]. Some studies have found that specific types of CAFs, such as tumorigenic CAFs, are correlated with poor prognosis, while others may be associated with better survival rates [15, 16, 18, 62, 63]. However, in LUAD, the functional diversity of CAFs and their prognostic relevance remain not fully understudied. Our work addresses this gap by systematically characterizing LAFs across disease stages, revealing nine subtypes, including the previously unrecognized nLAFs. While the proliferative gene signature of nLAFs aligns with the established pCAF subtype identified across multiple solid tumors [15, 20], our findings suggest additional context-specific features in LUAD. Beyond the shared expression of STMN1, TOP2A, and MKI67, nLAFs were observed to exhibit: (1) stage-dependent accumulation in advanced LUAD; (2) spatial proximity to hypoxic regions; and (3) correlative associations with immunosuppressive macrophage infiltration via putative COL1A1–CD44 signaling. Whether these features represent true functional specializations of pCAFs in lung tissue or merely reflect the distinct tumor microenvironment of LUAD remains to be determined through comparative functional studies. Nevertheless, these observations highlight the need for cancer type-specific characterization of fibroblast subpopulations rather than assuming universal functional equivalence.

Unlike mCAFs or iCAFs, which primarily influence ECM remodeling or immune suppression, nLAFs are distinguished by their proliferative gene signatures (e.g., STMN1, TOP2A) and enrichment in advanced LUAD. This aligns with recent pan-cancer studies identifying proliferative CAFs in ovarian and cholangiocarcinoma [15], yet our spatial transcriptomics data observed a higher ratio of nLAFs with LUAD patients at advanced stage, suggesting a spatially restricted role in LUAD aggressiveness. Furthermore, our nLRS model, derived from nLAF-specific markers, outperformed 49 existing prognostic signatures, underscoring its clinical novelty. While prior LUAD models focused on cell death, epithelial or immune markers [35, 64], our fibroblast-centric approach highlights stromal reprogramming as a critical prognostic axis.

Interestingly, NicheNet analysis identified HMGB2 as a key regulator of nLAF markers. HMGB2, a damage-associated molecular pattern (DAMP) protein, is released during cellular stress and activates NF-κB signaling in fibroblasts, fostering pro-inflammatory cytokine production (e.g., IL-6, TNF-α) [65]. In LUAD, HMGB2-driven nLAFs may amplify stromal-tumor crosstalk, enhancing ECM remodeling and immune evasion—a mechanism corroborated by elevated MDSC infiltration in nLRS-high patients.

In current study, the significant enrichment of nLAFs in advanced LUAD suggests a key role in tumor growth and metastasis. The scissor+ LAFs and nLAFs cell subsets identified by the scissor algorithm are closely associated with poor prognosis, potentially due to their promotion of tumor-related signaling pathways such as EMT and hypoxia within the TME. EMT is a hallmark of metastasis, driven by TGF-β and Wnt signaling [66]. We propose that nLAFs might secrete EMT-inducing factors (e.g., TGF-β1, SNAI1) to enhance tumor cell invasiveness. Concurrently, the enrichment of hypoxia-related genes in nLAFs suggest their role in stabilizing hypoxic niches, which promote angiogenesis and immunosuppression [67]. This dual functionality—pro-metastatic signaling and hypoxic TME shaping—may explain the poor prognosis of nLRS-high patients. Moreover, the high predictive performance of the nLRS model supports its potential clinical application, particularly in identifying high-risk LUAD patients and guiding personalized treatment.

Additionally, GSEA revealed glycolytic pathway activation in high-nLRS tumors, suggesting metabolic symbiosis between nLAFs and cancer cells. CAFs often exhibit a “reverse Warburg” phenotype, exporting lactate to fuel tumor growth while acidifying the TME to suppress immunity [68]. Such metabolic reprogramming may underlie the observed immunotherapy resistance in nLRS-high patients, as acidic microenvironments inhibit T-cell function [68]. Conversely, the inverse correlation between nLRS and chemotherapy sensitivity implies that nLAFs enhance drug resistance via ECM-mediated barrier effects, a hypothesis supported by studies in pancreatic cancer [69]. These results underscore the utility of nLRS as a biomarker for immunotherapy stratification and emphasize the importance of integrating nLRS with other predictive indices, such as MMR, to refine patient selection and optimize therapeutic efficacy. Notable, our analysis of immunotherapy response relies on pan-cancer cohorts due to the limited availability of large-scale LUAD-specific immunotherapy datasets with transcriptomic profiling. While the inverse correlation between nLRS and immunotherapy response appears consistent across cancer types, we emphasize that these findings cannot be directly extrapolated to LUAD without prospective validation. The biological mechanisms underlying immunotherapy resistance in high-nLRS tumors, such as ECM-mediated T cell exclusion and immunosuppressive macrophage recruitment, are likely conserved across cancer types [61], but their relative importance in LUAD specifically requires further investigation.

Within the nLRS panel, each of the five genes appears to contribute to LUAD biology through distinct but converging oncogenic processes. ALDOA, a key glycolytic enzyme, is frequently upregulated in LUAD and is linked to enhanced aerobic glycolysis, increased metastatic potential, and adverse prognosis; a recent mechanistic study showed that a pro‑metastatic tRNA fragment drives ALDOA oligomerization to boost glycolytic flux in LUAD cells, thereby supporting rapid proliferation and survival under metabolic stress and potentially fostering an immunosuppressive, lactate‑rich TME [70]. ANLN (anillin), a scaffold protein essential for cytokinesis, is markedly overexpressed in LUAD, where it promotes cell proliferation, invasion and cell‑cycle progression; functional knockdown of ANLN significantly suppresses lung cancer cell growth and migration, and multiple clinical and bioinformatics analyses have identified ANLN as an independent adverse prognostic marker that is also associated with altered immune cell infiltration and treatment response in LUAD [71, 72]. CKAP4, a type II transmembrane protein that serves as a receptor for DKK1, activates the PI3K/AKT pathway upon DKK1 binding and thereby enhances tumor cell proliferation and survival; in lung cancer, co‑expression of DKK1 and CKAP4 in tumor tissues and serum is associated with worse clinical outcomes, and CKAP4 has been proposed as both an exosomal diagnostic biomarker and a druggable surface target [73]. PRC1, a microtubule‑associated protein required for mitotic spindle organization and cytokinesis, is aberrantly upregulated in LUAD and has been shown in CRISPR and functional studies to drive tumorigenesis, proliferation, metastasis and lymph‑node spread, at least in part via activation of the Wnt/β‑catenin signaling pathway [74].

Interestingly, the SERPINH1 gene showed the highest specificity in our model, suggesting its potential as a biomarker for predicting lung cancer patient survival and guiding individualized treatment plans. SERPINH1 (HSP47), a top prognostic gene in the nLRS model, is associated with LUAD progression in our correlative analyses. While our in vitro experiments demonstrate that SERPINH1 overexpression promotes proliferation and migration in A549 cells (Fig. 12), whether SERPINH1 drives these phenotypes through collagen stabilization, immune modulation, or other mechanisms in vivo requires further investigation. Its role in collagen stabilization likely contributes to the fibrotic TME observed in nLRS-high patients, physically excluding cytotoxic T cells and fostering immune evasion—a phenomenon reported in multiple tumors [75]. High expression of SERPINH1 is associated with poor prognosis in gliomas and colorectal cancer and may also participate in the metastatic process of colorectal cancer by affecting immune infiltration and the TME [76]. Similar patterns of SERPINH1 expression have been observed in other tumor types, such as gastric cancer [77]. In LUAD, although research on SERPINH1 is not as extensive, existing studies hint at its possible modulation of the TME and intercellular interactions, thereby influencing tumor aggressiveness. Interestingly, SERPINH1 has been implicated in the crosstalk with CAFs, potentially facilitating tumor cell migration and invasion [78]. Beyond ECM effects, SERPINH1 correlated with genomic instability, possibly by impairing DNA repair through collagen-mediated oxidative stress [79]. This dual role-immune exclusion and mutagenesis-positions SERPINH1 as a central node in nLAF-mediated aggressiveness. However, a comprehensive understanding of SERPINH1‘s role in LUAD, particularly its prognostic value and how it may be leveraged in therapeutic strategies, remains to be further unexplored. Herein, we indicate that SERPINH1 may play a significant role in the development and progression of LUAD, making it a potential biomarker for prognosis prediction and guiding personalized therapy strategies.

By integrating multi-omics data across bulk transcriptomes, scRNA-seq, and spatial transcriptomes, and combining various machine learning algorithms, our study provides detailed insights into LAFs heterogeneity and its prognostic significance. Furthermore, our findings position nLRS as a actionable biomarker for personalized therapy. For instance, nLRS-high patients, resistant to immunotherapy, may benefit from specific chemotherapy agents. Conversely, nLRS-low patients with high TMB and MMR showed improved survival with immunotherapy.

Collectively, the nLRS model offers a novel tool for prognostic assessment in LUAD patients, aiding in the early identification of high-risk patients. By identifying drugs associated with nLRS, this study provides a fresh perspective on treatment options for LUAD patients, particularly in the realms of chemotherapy and targeted therapy.

## Limitations

Despite the promising findings of our study, several limitations should be noted. First, patient heterogeneity may affect the generalizability of the nLRS model. LUAD patients differ in genetic background, clinical features, and treatment exposure, which can influence model performance. Although we incorporated variables such as stage, smoking status, and age into our nomograms, more refined stratification and prospective validation in independent, well-annotated cohorts are still needed. Second, cohort-specific biases may impact the robustness of our conclusions. The datasets used here, while relatively diverse, may not fully represent the global LUAD population. Differences in inclusion criteria, sequencing platforms, and sample sizes across cohorts could introduce systematic bias. Future work should therefore include larger, multi-center cohorts from different regions and clinical settings to further validate and calibrate the nLRS. Third, although we have confirmed the protein expression of all model genes by Western blotting and immunohistochemistry, functional validation of the underlying mechanisms remains incomplete. The links between nLAF activity, nLRS, immune modulation, and LUAD progression are still largely based on correlative and computational evidence. Further in vitro and in vivo experiments, including genetic or pharmacologic perturbation of key genes (such as SERPINH1) and interrogation of COL1A1–CD44 signaling, are required to delineate causal pathways and evaluate their therapeutic potential. Similarly, while SERPINH1 overexpression promotes malignant phenotypes in vitro, its specific role in immune modulation and collagen stabilization in the LUAD microenvironment remains hypothetical. Finally, although spatial transcriptomics supported the spatial distribution and proportion of nLAFs in LUAD tissues, additional orthogonal validation is warranted. Future studies should incorporate flow cytometry (and potentially mass cytometry) to quantify nLAF abundance and phenotype across different disease stages and treatment contexts. Such multi-modal and longitudinal data will help to refine the nLRS model and better define its clinical utility.

## Conclusions

In this study, we integrated bulk, single‑cell, and spatial transcriptomic data to systematically characterize fibroblast heterogeneity in LUAD and identified nLAFs as a proliferative subtype associated with poor prognosis. Based on nLAF‑related genes, we developed and validated a five‑gene nLRS model that outperformed multiple published signatures and served as an independent prognostic factor across diverse cohorts. High nLRS was linked to an immunosuppressive and fibrotic microenvironment, reduced immunotherapy benefit, and distinct patterns of drug sensitivity, suggesting its utility for risk stratification and treatment decision‑making. Functional experiments further confirmed that SERPINH1 promotes proliferation and migration of NSCLC cells, supporting its potential as a therapeutic target. Together, these findings provide a fibroblast‑centered framework for prognosis and therapy optimization in LUAD, warranting further prospective and mechanistic validation.

## Electronic supplementary material

Below is the link to the electronic supplementary material.

Supplementary material 1

Supplementary material 2

Supplementary material 3

## Data Availability

Bulk RNA transcripts and scRNA-seq data used in this study are available from the TCGA Research Network portal, GEO, or the corresponding datasets. Spatial transcripts data are obtained from the E-MTAB-13530 cohort (https://www.ebi.ac.uk/biostudies/arrayexpress/studies/E-MTAB-13530). All data generated during this study are included in the manuscript and supporting files.

## Declarations

### Provenance and peer review

Not commissioned, externally peer-reviewed
