# Supplementary material for: Deciphering lung adenocarcinoma heterogeneity: a multi-omics approach reveals nuclear division fibroblasts as prognosticators and therapeutic targets
Source: J Transl Med. 2026 Mar 20;24:610. doi: 10.1186/s12967-026-08022-3 (PMC13126713; doi:10.1186/s12967-026-08022-3)
Supplement: Supplementary file 1 — Supplementary material 1 [file 12967_2026_8022_MOESM1_ESM.doc]

**Deciphering lung adenocarcinoma heterogeneity: a multi-omics approach reveals nuclear division fibroblasts as prognosticators and therapeutic targets**

Supplemental Figures

| 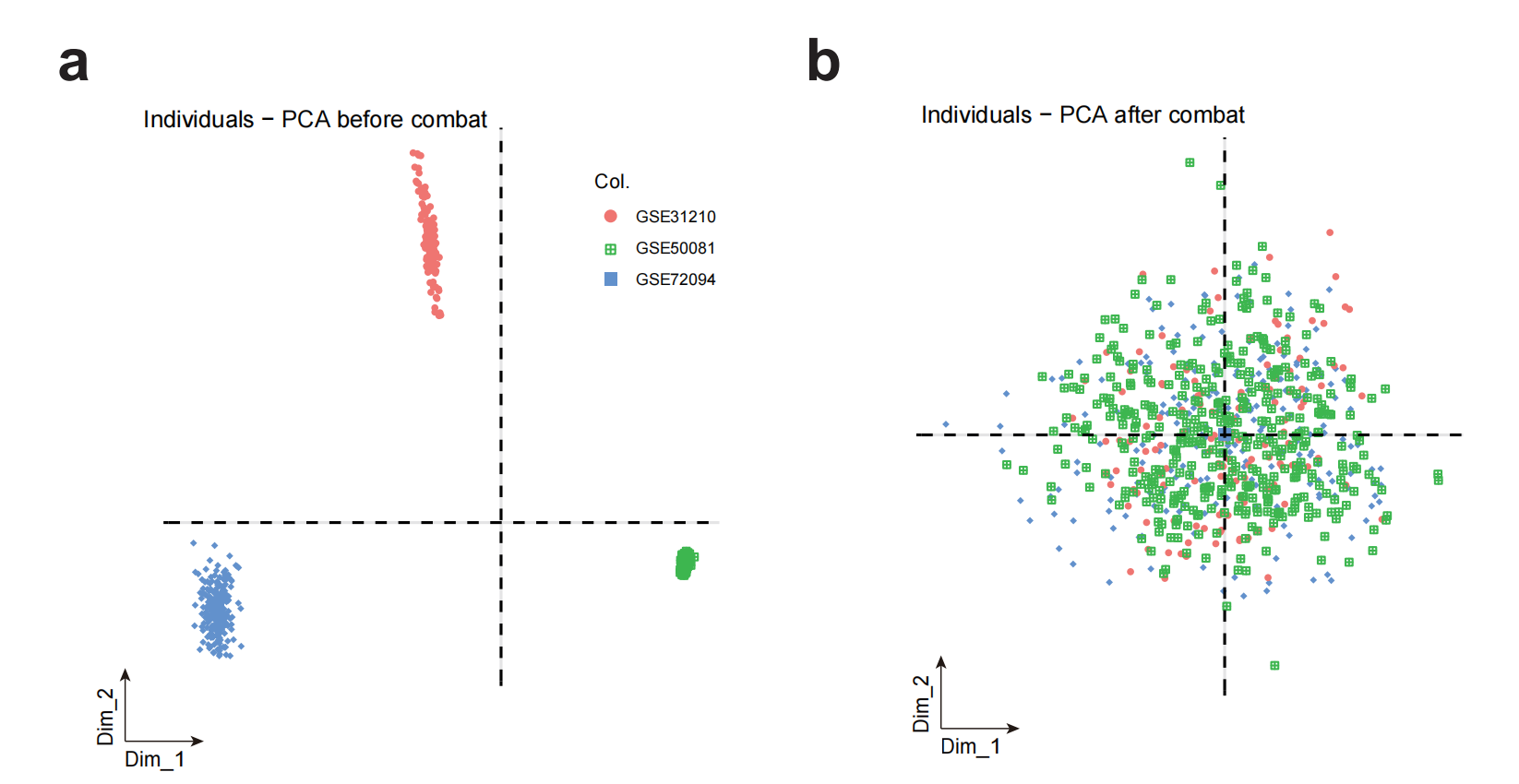 |
| --- |
| **Figure S1. Removed the batch effect of SVA algorithm in GSE31210, GSE50081, and GSE72094 cohorts.** (a) before combat. (b) after combat. |

| 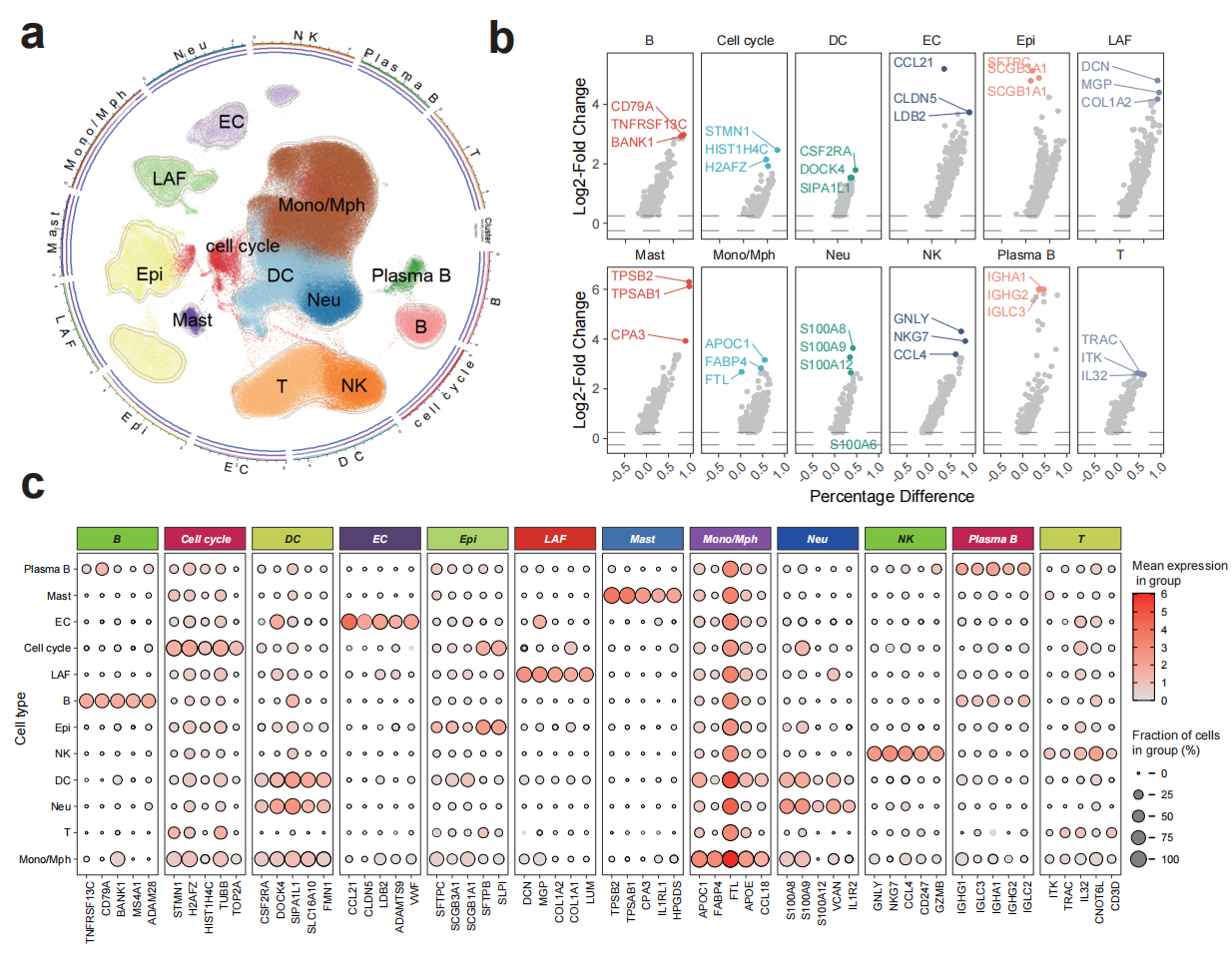 |
| --- |
| **Figure S2.** **Landscape of single-cell data from 93 samples and display of marker genes.** (a) Uniform manifold approximation and projection (UMAP) plot showing the major cell types. (b) The Top 3 significant marker genes of each cell types. (c) Doplot displaying the expression of specific marker genes in each cell types. |

| 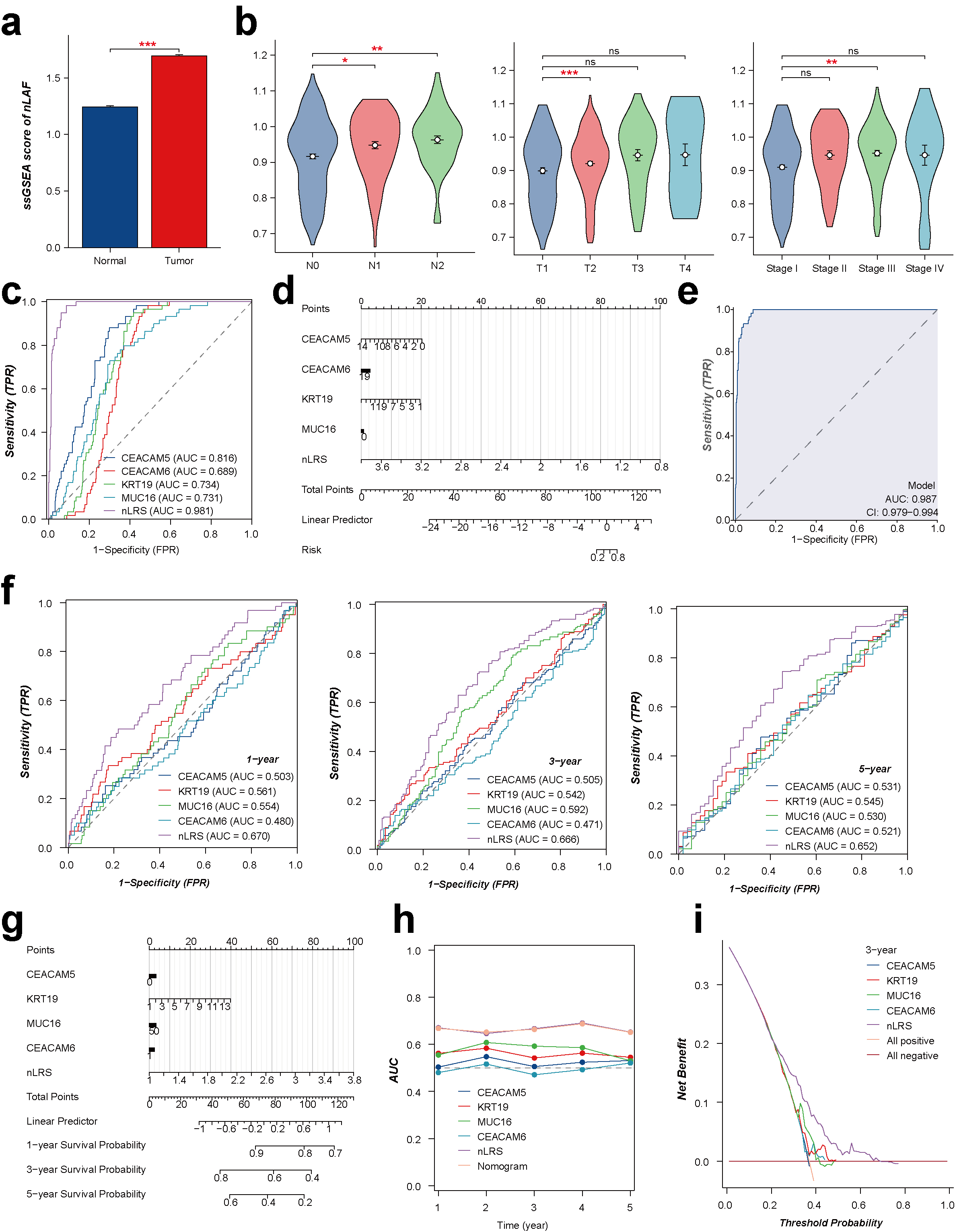 |
| --- |
| **Figure S3: Validation of nLAFs and Comparative Analysis of the nLRS Model.** (a-b) ssGSEA analysis of nLAF scores in TCGA-LUAD cohorts, demonstrating that higher nLAF scores are associated with the development and progression of LUAD. (c, f) Comparison of the nLRS model with traditional biomarkers (CEACAM5, CEACAM6, KRT19, MUC16) in LUAD diagnosis and prognosis. The nLRS model outperforms these biomarkers, indicating its superior predictive accuracy. (d, g) Construction of nomogram models integrating the nLRS model with traditional biomarkers for enhanced clinical decision support. (e) The combined model shows incremental value in LUAD diagnosis, enhancing diagnostic accuracy beyond traditional biomarkers alone. (h-i) No significant incremental value in prognosis is observed when combining the nLRS model with traditional biomarkers. |

| 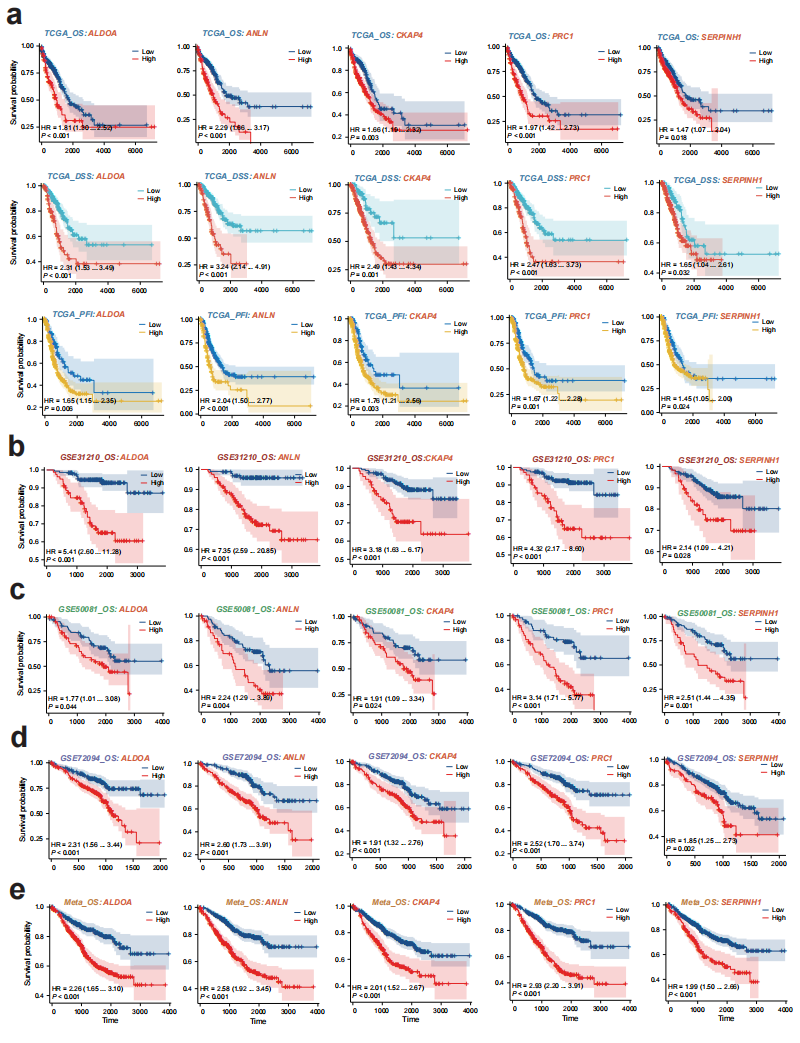 |
| --- |
| **Figure S4. Kaplan-Meier survival curves for each nLRS model genes across training and testing cohorts.** a-e showing the Kaplan-Meier survival curves for patients with low and high model gene expression from the TCGA, GSE31210, GSE50081, GSE72094, and meta cohorts. |

| 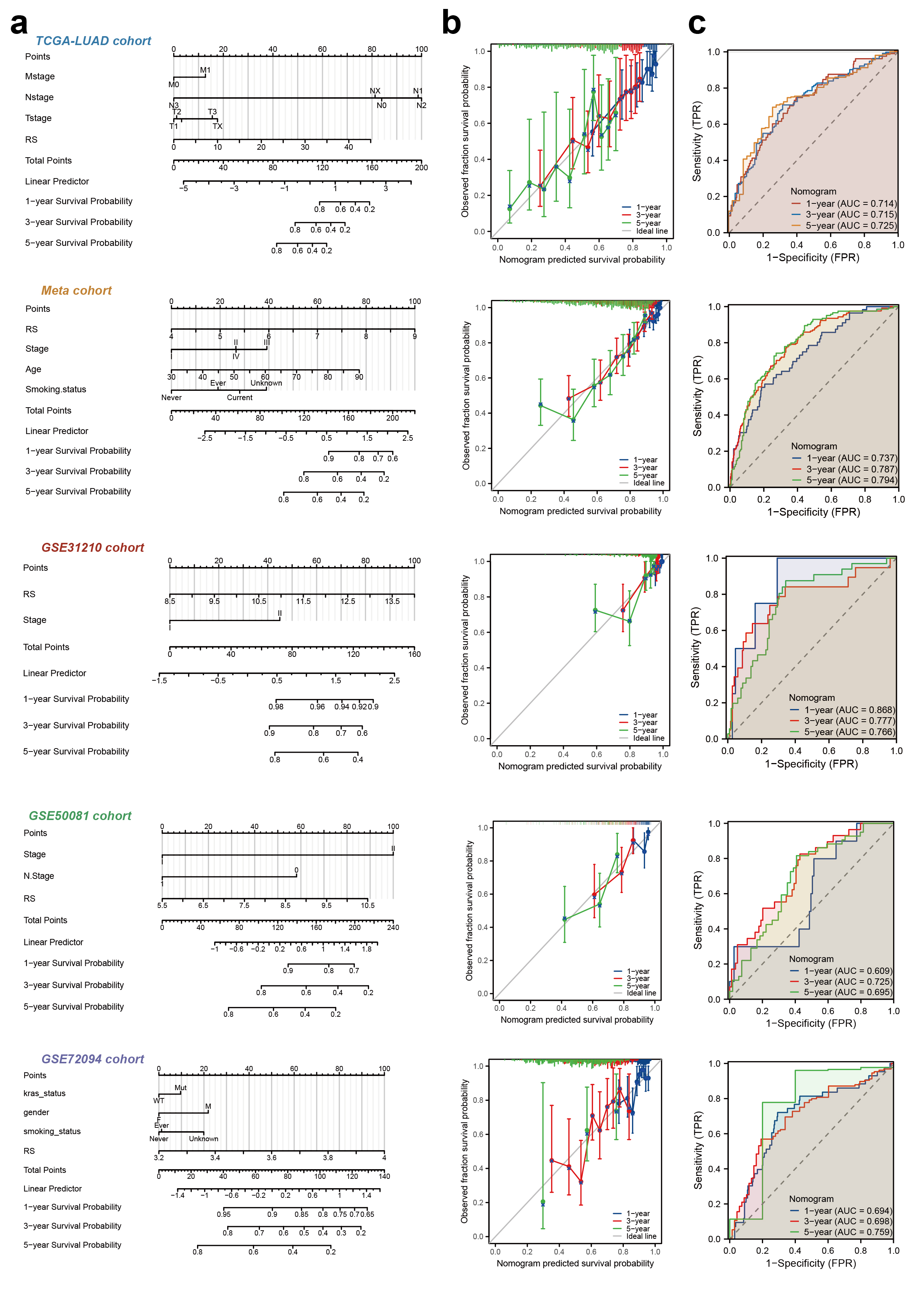 |
| --- |
| **Figure S5.** **Construction and evaluation of the prognostic nomogram model in training and testing cohort.** (a) Prognostic nomogram predicting the survival of patients in the TCGA, GSE31210, GSE50081, GSE72094, and Meta cohorts. (b) Calibration plots of nomogram-predicted 1-, 3-, and 5-year OS probabilities in he TCGA, GSE31210, GSE50081, GSE72094, and Meta cohorts. (C) Time-dependent ROC analysis of the nomogram for OS in The TCGA, GSE31210, GSE50081, GSE72094, and Meta cohorts. OS: Overall survival. |
| 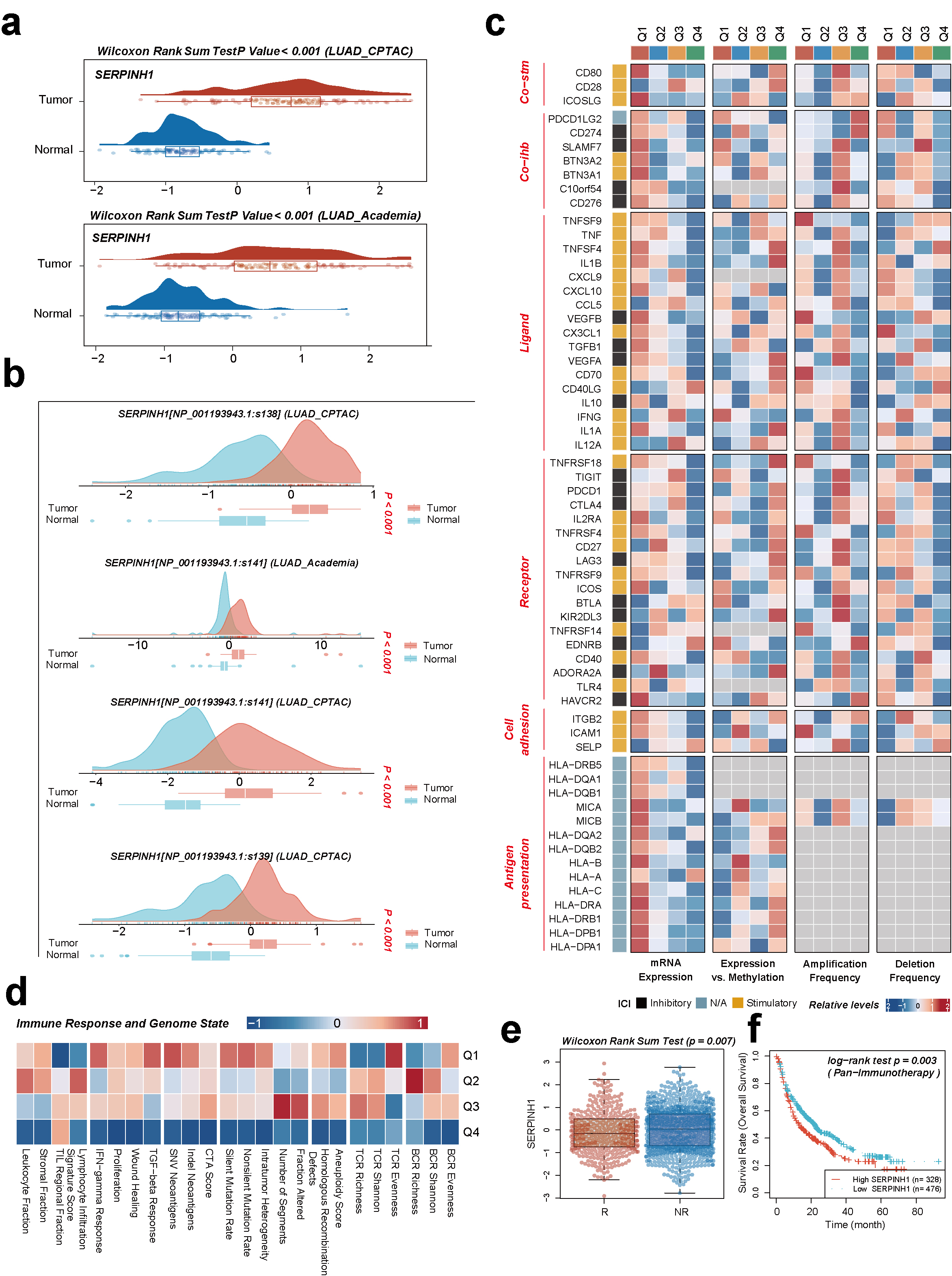 |
| **Figure S6. Figure S6: Comprehensive Analysis of SERPINH1 in LUAD.** (a) Differential expression of SERPINH1 protein levels in LUAD tissues compared to normal tissues, as evidenced by data from the CPTAC-LUAD (https://proteomic.datacommons. cancer.gov/pdc/study/PDC000153) and LUAD_Academia datasets (https://proteomic.datacommons.cancer.gov/pdc/study/ PDC000219). (b) Compare the expression level of the specific phosphorylation sites on SERPINH1 protein between the tumor/normal tissue. (c) Regulation of immunomodulatory molecules in the context of SERPINH1. Gene expression was divided into quartiles (Q1-Q4). The heatmap components, from left to right, represent mRNA expression (median-normalized expression levels), expression versus methylation (correlation with DNA methylation beta-values), amplification frequency (difference in amplification fraction between subtypes and all samples), and deletion frequency (difference in deletion fraction between subtypes and all samples). (d) Immune response and genomic state scores for subtypes Q1-Q4. The heatmap represents the intra-group mean of each score, standardized by row to ensure uniform scaling. (e-f) Correlation of high SERPINH1 expression with reduced sensitivity to immunotherapy and poorer clinical outcomes, as shown by data from pan-cancer immunotherapy cohorts. |
