# Supplementary figures and images for: Deciphering lung adenocarcinoma heterogeneity: a multi-omics approach reveals nuclear division fibroblasts as prognosticators and therapeutic targets
Source: J Transl Med. 2026 Mar 20;24:610. doi: 10.1186/s12967-026-08022-3 (PMC13126713; doi:10.1186/s12967-026-08022-3)

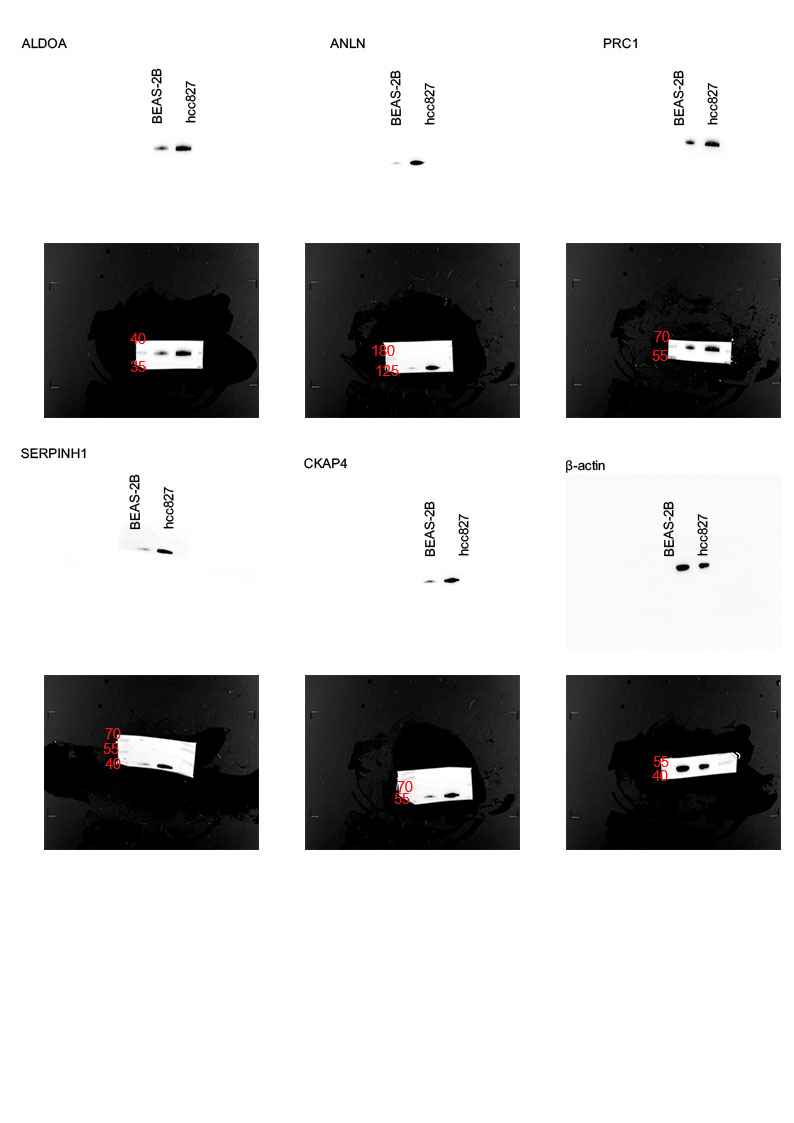


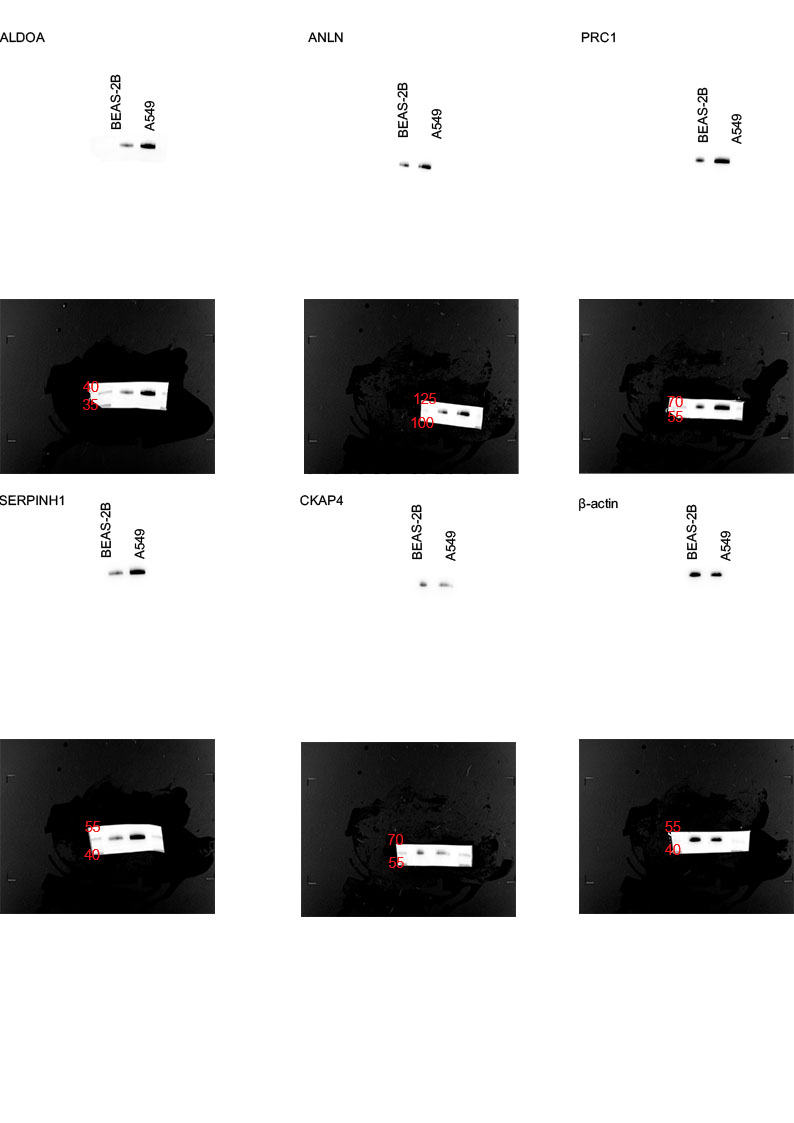


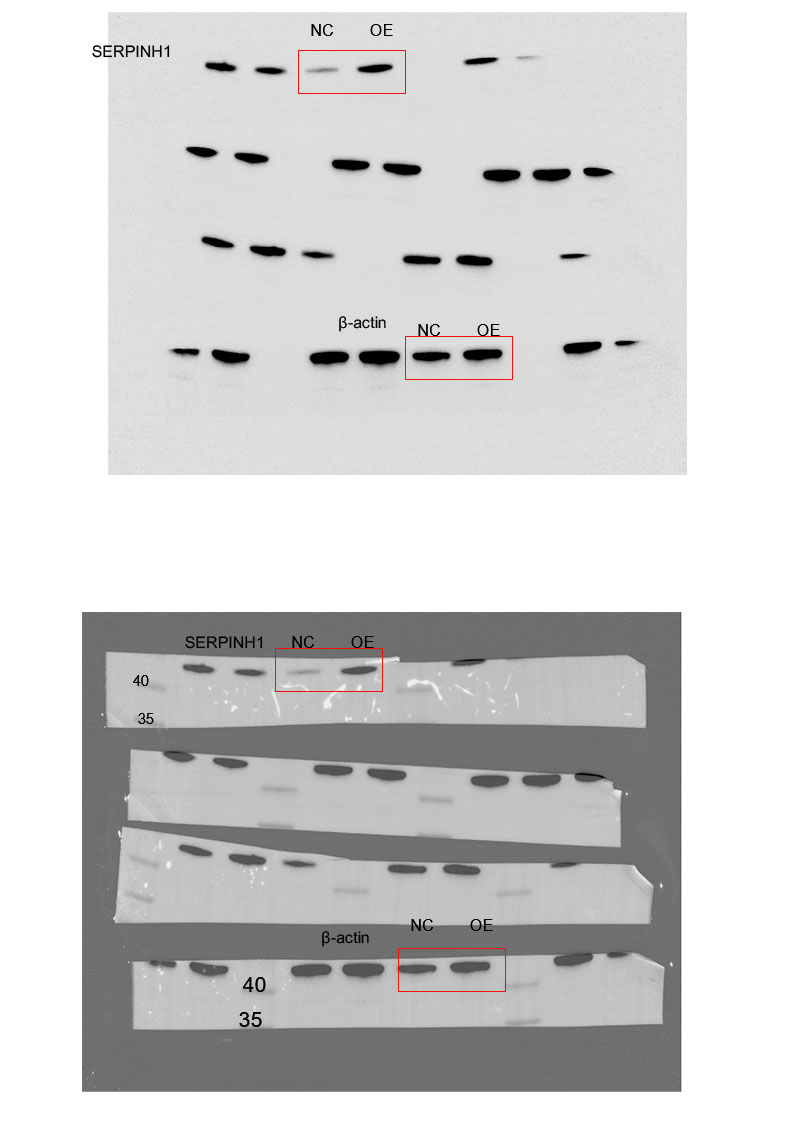

Supplement: Supplementary file 2 — Supplementary material 2 [file 12967_2026_8022_MOESM2_ESM.docx]
